# stAPAminer: Mining Spatial Patterns of Alternative Polyadenylation for Spatially Resolved Transcriptomic Studies

**DOI:** 10.1016/j.gpb.2023.01.003

**Published:** 2023-01-18

**Authors:** Guoli Ji, Qi Tang, Sheng Zhu, Junyi Zhu, Pengchao Ye, Shuting Xia, Xiaohui Wu

**Affiliations:** 1 Pasteurien College, Suzhou Medical College of Soochow University, Soochow University, Suzhou 215000, China; 2 Department of Automation, Xiamen University, Xiamen 361005, China; 3 Institute of Neuroscience, Soochow University, Suzhou 215000, China

**Keywords:** Alternative polyadenylation, Spatial transcriptomics, Single-cell RNA sequencing, Spatial pattern, Imputation

## Abstract

**Alternative polyadenylation** (APA) contributes to transcriptome complexity and gene expression regulation and has been implicated in various cellular processes and diseases. **Single-cell RNA sequencing** (scRNA-seq) has enabled the profiling of APA at the single-cell level; however, the spatial information of cells is not preserved in scRNA-seq. Alternatively, **spatial transcriptomics** (ST) technologies provide opportunities to decipher the spatial context of the transcriptomic landscape. Pioneering studies have revealed potential spatially variable genes and/or splice isoforms; however, the pattern of APA usage in spatial contexts remains unappreciated. In this study, we developed a toolkit called stAPAminer for mining **spatial patterns** of APA from spatially barcoded ST data. APA sites were identified and quantified from the ST data. In particular, an **imputation** model based on the k-nearest neighbors algorithm was designed to recover APA signals, and then APA genes with spatial patterns of APA usage variation were identified. By analyzing well-established ST data of the mouse olfactory bulb (MOB), we presented a detailed view of spatial APA usage across morphological layers of the MOB. We compiled a comprehensive list of genes with spatial APA dynamics and obtained several major spatial expression patterns that represent spatial APA dynamics in different morphological layers. By extending this analysis to two additional replicates of the MOB ST data, we observed that the spatial APA patterns of several genes were reproducible among replicates. stAPAminer employs the power of ST to explore the transcriptional atlas of spatial APA patterns with spatial resolution. This toolkit is available at https://github.com/BMILAB/stAPAminer and https://ngdc.cncb.ac.cn/biocode/tools/BT007320.

## Introduction

Messenger RNA (mRNA) polyadenylation is a critical mRNA processing event that occurs toward the completion of transcription and involves two tightly coupled steps: cleavage at the nascent transcript followed by the addition of an untemplated poly(A) tail to its 3′ end [1], [2]. Over 70% of mammalian genes possess more than one poly(A) site, suggesting the possibility of the modulated use of selective poly(A) sites through alternative polyadenylation (APA) [1], [2]. APA contributes substantially to the complexity of the transcriptome and proteome by generating isoforms of the same gene with distinct 3′ untranslated regions (UTRs) or coding regions. APA is dynamically regulated in various cellular processes and diseases, including cell activation, proliferation, differentiation, neurodegenerative disorders, and cancer [3], [4], [5], [6], [7], [8], [9], [10], [11], [12]. Studies using bulk 3′ end sequencing (3′-seq) [2], [13], [14] and/or RNA sequencing (RNA-seq) [14], [15] revealed extensive 3′ UTR lengthening or shortening events in various processes. For example, 3′ UTRs generally shorten in proliferating cells, whereas 3′ UTRs lengthen during embryonic differentiation [16] and animal neurogenesis [17], [18].

In contrast to bulk methodologies, single-cell RNA sequencing (scRNA-seq) protocols characterize the transcriptional landscape in individual cells, with many protocols utilizing 3′ selection or enrichment in library construction, such as Drop-seq [19], CEL-Seq [20], and 10X Genomics [21]. Although most scRNA-seq studies initially focus only on gene expression profiling, these scRNA-seq technologies inherently capture a notable amount of information on isoform usage, providing substantial potential to profile APA events at the single-cell level. Computational tools, including scDAPA [22], scAPA [23], Sierra [24], scAPAtrap [25], and scDaPars [26], have been proposed to identify APA sites in single cells and/or profile differential APA isoform usage among cell types using scRNA-seq. Particularly, single-cell APA profile compiled from scRNA-seq enables the discovery of hidden subpopulations of cells that are unrecognizable in conventional gene expression analysis [25], [26], revealing the possibility of discerning cell identities with the APA layer independent of gene expression.

Even though scRNA-seq is powerful in profiling the transcriptome of individual cells, the spatial information of cells is not preserved because of tissue dissociation prior to sequencing. The characterization of the spatial organization and molecular features of cells is essential to understanding cellular interactions and organization in the tissue microenvironment [27]. Several strategies for spatial transcriptomics (ST), including MERFISH [28], seqFISH [29], and ST through spatial barcoding [30], have been established to measure spatially resolved gene expression, and they provide opportunities to decipher the spatial context of the transcriptomic landscape within single cells and/or across tissue sections. The identification of spatially variable (SV) genes is usually the first critical step in analyzing ST data to spatially resolve the transcriptomic landscape across tissues. Recently, a few computational approaches have been proposed to explore spatial gene expression trends, including SpatialDE [31], Trendsceek [32], SPARK [33], and SPARK-X [34]. Continuous gradients or spatial gene expression patterns can be identified by these tools, and this contributes to disclosing significant biological discoveries that otherwise cannot be revealed using scRNA-seq alone. Although most spatially resolved transcriptomic studies have restricted analysis at the gene level, these studies, particularly the ST method of spatial barcoding, may provide additional information on transcript isoforms, enabling multiple layers of transcriptome information to be obtained from ST experiments without changing the experimental methods. Recently, dynamic alternative splicing and brain region-specific isoform expression have been observed using single-cell isoform RNA sequencing technology [35]. These pioneering studies have implicated potential SV genes and/or splice isoforms. However, the pattern of APA usage in spatial contexts remains unclear.

In this study, we developed a toolkit referred to as stAPAminer for mining spatial patterns of APA from spatially barcoded ST data. First, poly(A) sites were identified from ST data, and then APA site usages of genes in individual spots were quantified. In particular, an imputation model based on k-nearest neighbors (KNN) was designed to recover APA signals obtained from the ST data by borrowing information from the spatial gene expression profile, and this can effectively mitigate the noise and bias caused by the dropout phenomenon. Based on the profile of imputed APA usage, APA genes with differential APA usage between morphological layers and genes with global spatial trends in APA usage variation were identified. By analyzing well-established mouse olfactory bulb (MOB) ST data, we presented a detailed view of spatial APA usage across MOB regions with stAPAminer. We compiled a comprehensive list of genes with spatial APA dynamics and obtained several major spatial APA patterns that represent APA dynamics in different morphological layers. These genes were enriched in Gene Ontology (GO) terms directly associated with olfactory bulb development, highlighting the benefits of spatial APA analysis using stAPAminer. By extending the analysis to two additional replicates of the MOB ST data, we observed that the spatial APA patterns of several genes were reproducible among replicates, thus demonstrating the robustness and effectiveness of stAPAminer in spatial APA analysis. stAPAminer employs the power of ST to explore the transcriptional atlas of spatial APA patterns with spatial resolution variation, establishing an additional layer of gene expression at isoform resolution.

## Results

### stAPAminer facilitates the analysis of spatial APA dynamics from ST data

**Figure 1:**
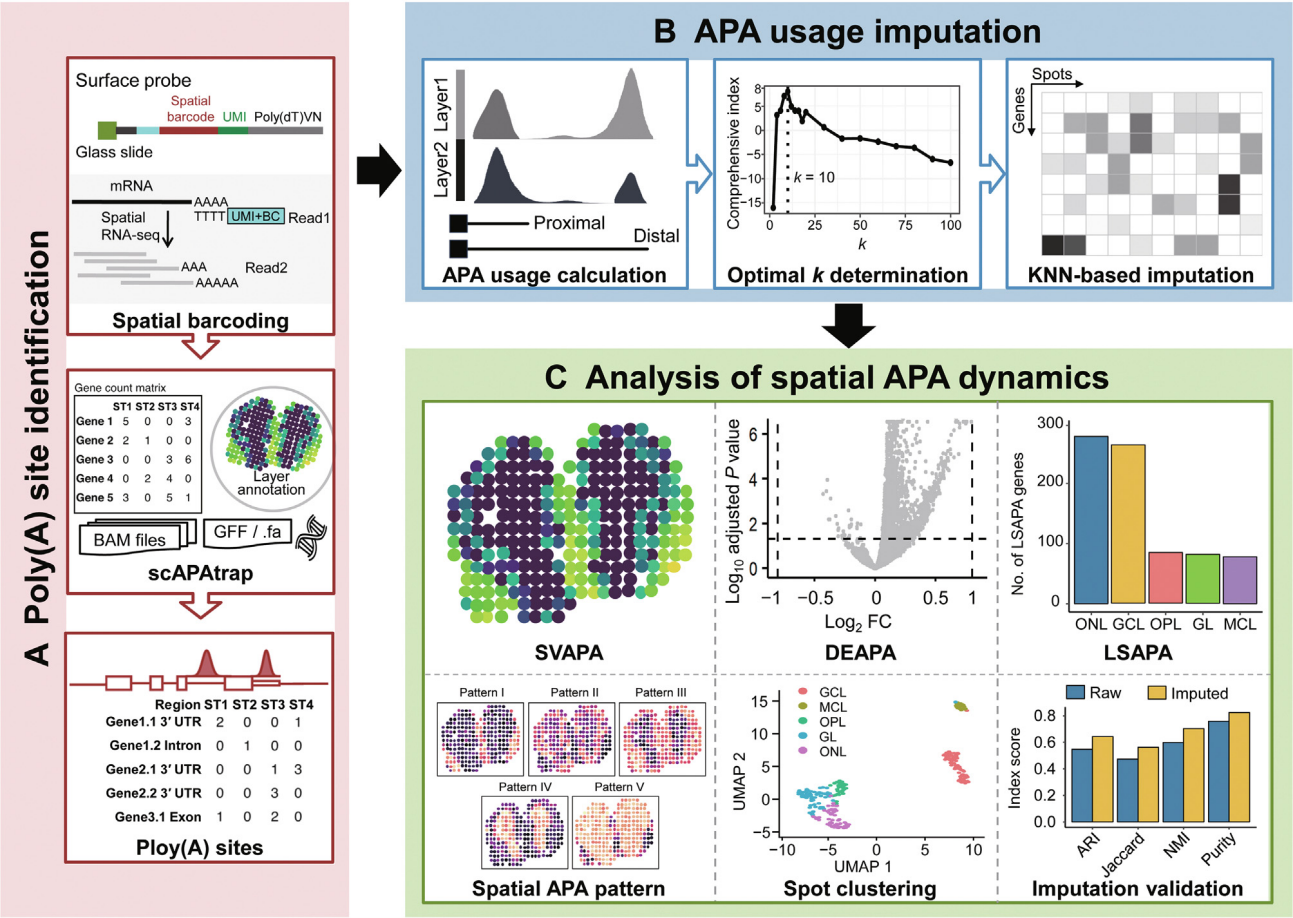
**Schema of stAPAminer** **A.** Identification of poly(A) sites from ST data using scAPAtrap. **B.** Quantification and imputation of APA usages. The sparse gene–spot matrix of APA usages is imputed by the KNN-based imputation model embedded in stAPAminer and the optimal *k* value is determined using a comprehensive index. **C.** Analysis of spatial APA dynamics with stAPAminer. stAPAminer can be used to detect SVAPA, DEAPA, and LSAPA. SVAPA, spatially variable APA usage; DEAPA, differential APA usage between layers; LSAPA, layer-specific DEAPA; mRNA, messenger RNA; RNA-seq, RNA sequencing; ST, spatial transcriptomics; UMI, unique molecular identifier; BC, barcode; UTR, untranslated region; GCL, granular cell layer; MCL, mitral cell layer; OPL, outer plexiform layer; ONL, olfactory nerve layer; GL, glomerular layer; ARI, adjusted rand index; NMI, normalized mutual information; UMAP, Uniform Manifold Approximation and Projection; BAM, Binary Alignment/Map; GFF, General Feature Format; APA, alternative polyadenylation; FC, fold change; KNN, *k*-nearest neighbors.

We developed an R package, stAPAminer, to explore spatial APA dynamics from ST data (Figure 1). First, poly(A) sites from ST data were identified and quantified using existing tools such as scAPAtrap (Figure 1A). Subsequently, a poly(A) site expression matrix was obtained, and this was similar to the conventional gene–cell expression matrix obtained from scRNA-seq, except that each row denotes one poly(A) site rather than a gene and each column denotes a spot. Next, a matrix of 3′ UTR or intronic APA usage was obtained from the poly(A) site expression matrix (Figure 1B). Genes with at least two 3′ UTR poly(A) sites were extracted for 3′ UTR APA analysis, and the APA usage of each gene was represented by the relative usage of the distal poly(A) site (RUD). For intronic APA analysis, APA genes with at least one intronic poly(A) site were extracted, and the APA usage of each gene was represented by the ratio of the intronic site (see Materials and methods). The APA usage matrix was even sparser than the gene–spot expression matrix. Therefore, we proposed a KNN-based imputation model for recovering APA signals in the APA usage matrix. This model leverages the gene expression profile to infer the spot–spot distance and then imputes the sparse APA usage matrix by borrowing information on APA usage from neighboring spots. The *k* value is the most critical parameter in the KNN-based imputation model, which can be determined using a strategy based on a comprehensive index (see Materials and methods). After the imputation, benchmarking analyses were conducted to investigate the performance of the imputation model. The imputed APA usage matrix was used to explore spatial APA dynamics (Figure 1C) to detect genes with differential APA usages between layers (DEAPA), layer-specific DEAPA (LSAPA), and SV APA usages (SVAPA). These three APA genes reflect the spatial characteristics of APA from different aspects, establishing the full landscape of spatial APA dynamics. Moreover, representative spatial APA patterns can be obtained by clustering these APA genes based on their APA usage profiles. stAPAminer was implemented as an open-source R package available at https://github.com/BMILAB/stAPAminer.

### Genome-wide poly(A) sites were identified from MOB ST data

**Figure 2:**
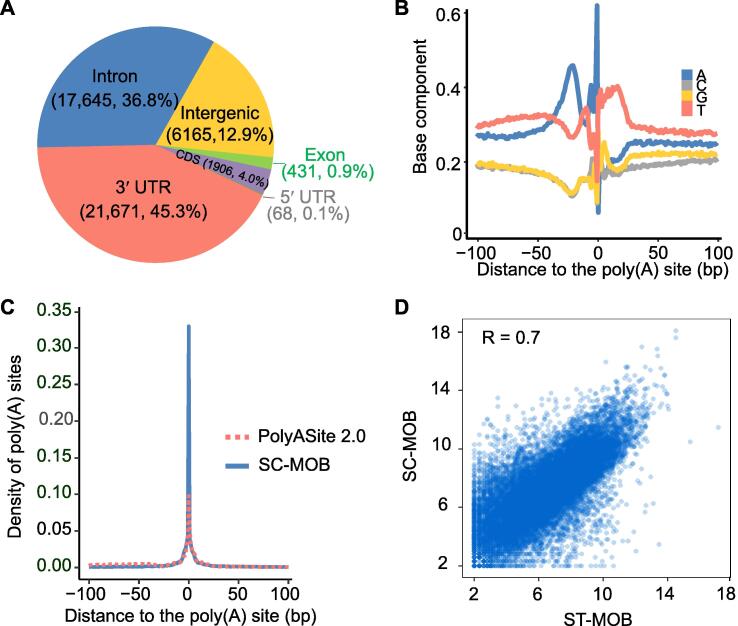
**Validation of poly(A) sites identified from the ST-MOB data** **A.** Distribution of poly(A) sites from ST-MOB in different genomic regions. **B.** Nucleotide compositions of the sequences surrounding 3′ UTR poly(A) sites from ST-MOB. Y-axis denotes the fractional nucleotide content at each position. On the X-axis, “0” denotes the poly(A) site. **C.** Comparison of the ST-MOB poly(A) sites with the annotated poly(A) sites in the PolyASite 2.0 database and the poly(A) sites identified from SC-MOB. The curves show the distance from ST-MOB poly(A) sites to annotated poly(A) sites and SC-MOB poly(A) sites. **D.** Scatter plot showing the correlation of poly(A) site expression profiles obtained from ST-MOB and SC-MOB. Each dot is one poly(A) site and the axis is log_2_ scaled. Pearson’s correlation is indicated in (D). CDS, coding sequence; MOB, mouse olfactory bulb; scRNA-seq, single-cell RNA sequencing; SC-MOB, scRNA-seq data of MOB.

We applied stAPAminer to analyze a spatially barcoded ST dataset of MOB [30]. Following other studies [31], [33], we primarily used the MOB Replicate 11 file for analysis, which consists of 11,274 genes on 260 spots after filtration (hereafter referred to as ST-MOB). A total of 47,886 poly(A) sites were extracted using the scAPAtrap pipeline (Figure 2A), with an average of 14,436 poly(A) sites per spot (Table S1). The majority of the poly(A) sites were located in 3′ UTRs/extended 3′ UTRs (21,671) or introns (17,645), and this is consistent with previous observations in scRNA-seq [24], [25]. The base composition surrounding the 3′ UTR poly(A) sites from ST-MOB resembled the general profile of annotated poly(A) sites (Figure 2B). A high frequency of core poly(A) hexamers was observed in the upstream poly(A) site region, including AAUAAA, AUUAAA, and GU-rich hexamers (Figure S1). Next, we used the poly(A) sites identified from MOB scRNA-seq data (hereafter referred to as SC-MOB) [36] and annotated poly(A) sites of bulk 3′-seq from PolyASite 2.0 [37] to validate the poly(A) sites identified from ST-MOB. Up to 16,433 poly(A) sites and 28,290 poly(A) sites from ST-MOB were detected in SC-MOB and PolyASite 2.0, respectively. In contrast, 24,891 poly(A) sites were exclusively observed in ST-MOB, which may represent potential novel polyadenylation events in the ST data that cannot be detected using scRNA-seq or bulk 3′-seq. Poly(A) sites from ST-MOB were close to the annotated poly(A) sites or sites from SC-MOB (Figure 2C). Additionally, the poly(A) site expression profile of ST-MOB was highly correlated with that of SC-MOB (Pearson’s correlation = 0.7) (Figure 2D) and neural-related samples from bulk 3′-seq (Pearson’s correlation = 0.69) (Figure S2). These observations demonstrate the authenticity of the poly(A) sites identified from ST-MOB data.

### stAPAminer effectively recovers highly sparse APA signals obtained from ST-MOB

**Figure 3:**
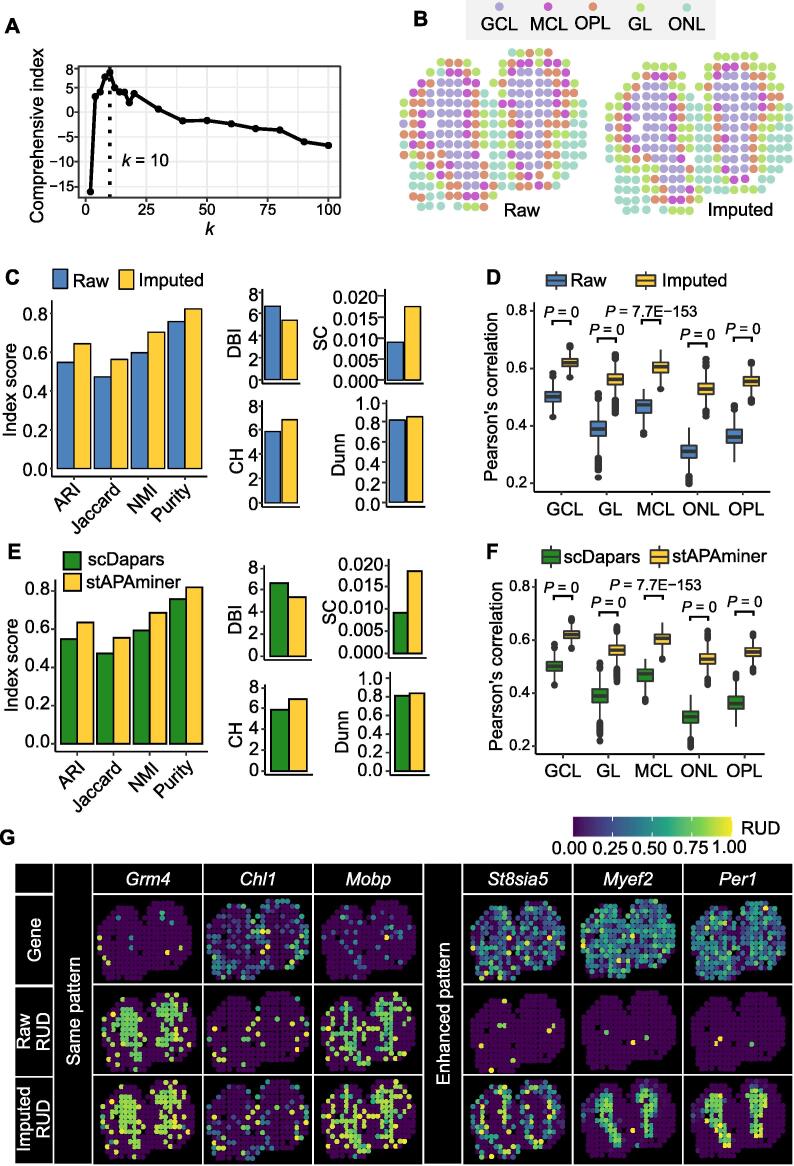
**Validation of stAPAminer in imputing APA signals** **A.** The comprehensive index score with the increase in the *k* value. The vertical dotted line marks the optimal *k* value (*k* = 10), corresponding to the maximum comprehensive index score. **B.** Visualization of ST spots on the tissue image before (raw) and after (imputed) imputation. **C.** Evaluation of the performance of the imputation model. Four metrics were used for evaluating the performance in the context of clustering, including ARI, Jaccard, NMI, and Purity, and four internal validation metrics without relying on the reference labels were also used, including DBI, CH, SC, and Dunn. **D.** Boxplot showing Pearson’s correlations between spot pairs in each layer estimated using imputed and the raw RUD scores. For each layer, correlations of all pairwise spots were calculated. **E.** Evaluation of the performance of stAPAminer and scDaPars. **F.** Boxplot showing Pearson’s correlations between spot pairs in each layer estimated between stAPAminer and scDaPars. **G.** Spatial patterns of APA usages for representative genes using raw (middle) and imputed (bottom) RUD matrices. The top row shows the tissue image based on the scaled gene expression level of the respective gene. Color represents RUD scores or scaled gene expression levels (yellow, high; blue, low). RUD, relative usage of the distal poly(A) site; DBI, Davies and Bouldin index; CH, Calinski–Harabasz; SC, silhouette coefficient.

After identifying poly(A) sites from ST-MOB, the expression level of each poly(A) site was quantified by counting the effective reads of the respective poly(A) site region (*i.e.*, peak). Here, we analyzed the 3′ UTR APA genes. For genes with multiple poly(A) sites in the 3′ UTR, RUD score (between 0 and 1) was used to measure the global trend of the 3′ UTR length change. An RUD matrix was generated from ST-MOB data, with rows representing genes and columns representing spots. To mitigate the impact of the high dropout rate, we proposed a KNN-based imputation method implemented in our stAPAminer package to recover the RUD matrix. First, we examined the clustering performance based on the comprehensive index for different *k* values from 2 to 100 and determined that the optimal *k* value for the KNN model was 10 (Figure 3A). After imputation with the KNN model (*k* = 10), we examined whether stAPAminer could efficiently recover the profile of APA usages. We applied Uniform Manifold Approximation and Projection (UMAP) visualization to the raw and imputed RUD matrices to show the differences between MOB layers. The 2-dimensional embeddings showed that the different layers became considerably distinguishable after the imputation (Figure 3B). The inferred layer labels of spots based on the imputed RUD matrix were more consistent with the reference labels than those inferred from the raw RUD matrix [*e.g.*, adjusted rand index (ARI): imputed = 0.643; raw = 0.547] (Figure 3C). Similar results were obtained using three other metrics, namely Jaccard, normalized mutual information (NMI), and Purity. Four additional metrics without relying on the reference labels, namely the Davies and Bouldin index (DBI) [38], Calinski–Harabasz (CH) [39], silhouette coefficient (SC) [40], and Dunn index [41], were used to quantitatively assess spot separation. According to these metrics, the imputed RUD matrix improved spot separation by recovering the true signals of APA usage (Figure 3C). We further compared the spot–spot correlation in the same layer of the tissue section using the imputed and raw RUD matrices. The median Pearson’s correlation of spot pairs in the same layer was only 0.311 to 0.501 using the raw RUD matrix, whereas stAPAminer substantially increased the spot–spot correlation of all five layers (from 0.525 to 0.622) (Figure 3D). Previously, scDaPars [26] was proposed to identify and quantify APA events from scRNA-seq data. It first identifies APA events from scRNA-seq using DaPars [8], a tool for identifying APA events from bulk RNA-seq, and then utilizes a regression model to impute the missing values in the APA usage matrix obtained from scRNA-seq. Although the underlying strategies differ, both stAPAminer and scDaPars can impute sparse APA signals. Here, we used scDaPars to impute raw APA signals obtained from ST-MOB and then compared the performances of scDaPars and stAPAminer. Regardless of the performance indicators used, stAPAminer outperformed scDaPars (Figure 3E and F), further demonstrating its superiority in imputing highly sparse APA signals.

Next, we examined whether stAPAminer could reveal spatial APA usage patterns. We used SPARK [33] to identify genes with SVAPA (see Materials and methods). For genes with strong SVAPA (*e.g.*, the well-known layer-specific marker genes *Grm4* [42], *Chl1* [43], and *Mobp* [44]), the pattern from imputed data was concordant with that from the raw data (Figure 3G). Notably, the patterns of these genes were considerably more distinguishable according to the APA profile (either RUD or imputed RUD) than according to the raw gene spot expression profile. In particular, we also observed cases wherein the raw RUD matrix exhibited a substantially weak signal that was enhanced in the imputed data (*e.g.*, *St8sia5*, *Myef2*, and *Per1*) (Figure 3G). For example, *St8sia5* was highly expressed in all layers, whereas no pattern was observed according to either the gene expression profile or the raw RUD profile. In contrast, after imputation, a distinct pattern was observed for this gene, with much higher RUD scores (*i.e.*, longer 3′ UTR) on the mitral cell layer (MCL) or outer plexiform layer (OPL) than on the other layers. Two 3′ UTR poly(A) sites were identified in ST-MOB for *St8sia5* (Table S1), both of which were supported by annotated poly(A) sites from PolyA_DB 3 [45]. *St8sia5* has been reported to induce ganglioside GQ1b expression and enhance neuronal differentiation via the MAP kinase pathway [46]. Another gene, *Myef2*, is a transcriptional repressor of the myelin basic protein gene, the expression of which is usually up-regulated in nerve sheath myxomas and schwannomas [47]. In addition, gene has two 3′ UTR poly(A) sites identified from ST-MOB, both of which were annotated in PolyA_DB 3. *Myef2* has a unique pattern according to the imputed RUD profile, with a longer 3′ UTR on the granular cell layer (GCL) than on the other layers. A similar case was observed for *Per1*, which contributes to phasing molecular and electrical circadian rhythms in suprachiasmatic nucleus neurons to increase the robustness of cellular timekeeping [48]. These results show that the imputation strategy in stAPAminer can effectively mitigate the noise caused by the dropout phenomenon and help detect genes with distinct patterns of APA usage from highly sparse and noisy ST data.

### stAPAminer reveals spatial dynamics of 3′ UTR APA usages from ST-MOB

**Figure 4:**
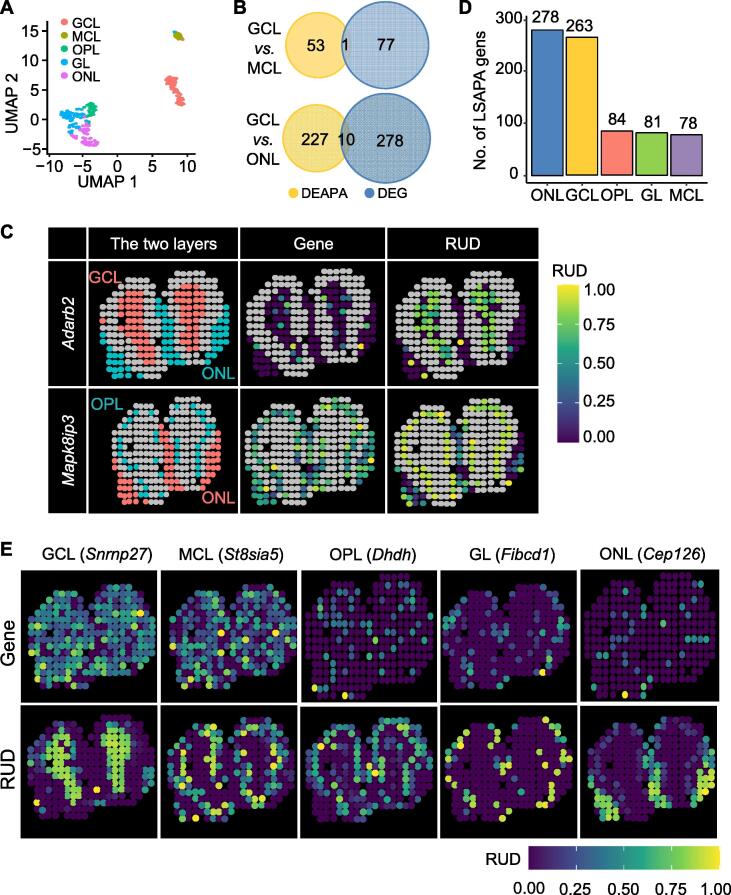
**Application of stAPAminer to ST-MOB data for identification and analysis of genes with spatial APA pattern** **A.** UMAP plot showing the 2-dimensional embeddings of spots on the five layers. **B.** Venn diagrams showing the overlap between DEAPA genes and DEGs by comparing GCL and MCL (top) as well as GCL and ONL (bottom). **C.** Two representative genes showing clear spatial APA usage patterns but no gene expression patterns. *Adarb2* is a DEAPA gene between GCL and ONL; *Mapk8ip3* is a DEAPA gene between OPL and ONL. Gene expression levels and RUD scores of spots on the respective layers are shown, and spots on other layers are colored as gray. **D.** Number of LSAPA genes on each layer. **E.** Representative LSAPA genes on the five layers, which showed clear spatial APA usage patterns but no gene expression patterns. DEG, differentially expressed gene.

Based on the imputed gene–spot RUD matrix, we explored APA usage profiles in spatially defined domains within the olfactory bulb. The RUD profile clearly separated the different morphological layers (Figure 4A). We detected 905 genes (403 non-redundant genes) with differential APA usage (*i.e.*, DEAPA genes) between each pair of morphological layers (Table S2). For comparison, we also identified 1146 differentially expressed genes (DEGs) using only the gene–spot expression matrix (Table S3). Although the numbers of DEAPA genes and DEGs were comparable, the overlap between the two gene sets was substantially limited, and a considerable number of genes were exclusively present in the DEAPA gene list. For example, between the GCL and MCL, only one common gene was observed among 78 DEGs and 54 DEAPA genes (Figure 4B). A similar case was observed in the GCL and olfactory nerve layer (ONL). These DEAPA genes were not recognizable by conventional gene expression analysis but represented genes with differential APA usage between layers. Among the 905 DEAPA genes, 111 were not present in the conventional gene–spot expression matrix. This may be because the scAPAtrap that we used was highly sensitive in capturing poly(A) sites, even for minimally expressed genes, whereas these genes may not be detected in conventional gene expression analysis pipelines. Figure 4C shows two representative example genes, which showed clear spatial APA usage patterns but no gene expression patterns. *Adarb2* (also known as *ADAR3*) was extremely low and loosely expressed in GCL or ONL according to the gene expression profile, and no pattern was observed between these two layers. In contrast, it has two 3′ UTR poly(A) sites exclusively identified from ST-MOB (Table S1), showing differential APA usage between GCL and ONL. *Adarb2* encodes a catalytically inactive protein that is primarily expressed in the brain, thalamus, and amygdala, and may be associated with disorders such as amyotrophic lateral sclerosis [49]. *Mapk8ip3* encodes a protein that functions as a motor in brain neurons, moving items along the axons [50]. *Mapk8ip3* was moderately expressed in both OPL and ONL without differential gene expression. Two 3′ UTR poly(A) sites were identified for this gene (Table S1), and differential APA usage was observed between OPL and ONL. Therefore, the APA profile potentially encodes complementary information that is absent or insignificant in the conventional gene–spot expression profile, which contributes to distinguishing the morphological layers. Next, we examined the presence of these DEAPA genes among known biologically important genes in the olfactory system. We compiled representative genes in the olfactory system presented in previous studies and public resources (Table S4), including marker genes highlighted in the original study [30], cell type-specific marker genes provided in a recent scRNA-seq study on MOB [36], and genes related to the olfactory system from the Harmonizome database [51]. A moderate number of DEAPA genes (71 out of 403 non-redundant genes) were present in the representative gene list, suggesting the potential biological function of these APA genes. Indeed, the overlap is relatively limited; however, this is not unexpected as these DEAPA genes were identified based on spatial APA profiles independent of gene expression, whereas the compiled representative gene list is based on gene expression profiles.

Furthermore, we detected 784 LSAPA genes by comparing the RUD profile of one layer to all other layers (Table S5). The majority of these LSAPA genes (69%) were detected in the GCL and ONL (Figure 4D). Figure 4E shows some representative LSAPA genes that demonstrated clear spatial APA usage patterns but no gene expression patterns. For example, *Snrnp27* and *St8sia5* are constitutively expressed across all layers; however, they show distinct spatial APA usage patterns on GCL and MCL, respectively. *Snrnp27* has two poly(A) sites identified from ST-MOB (Table S1), and a previous microarray study identified altered expression of this gene during the progression of Alzheimer’s disease [52]. Additionally, *Fibcd1*, a known chitin-binding receptor of the innate immune system, shows glomerular layer (GL) specificity according to the gene expression and APA profiles. However, the layer-specific pattern obtained from the APA profile was more distinguishable. *Fibcd1* has recently been recognized as an evolutionarily conserved component of the brain extracellular matrix that is associated with a complex neurodevelopmental disorder [53]. Notably, two other genes, *Dhdh* and *Cep126*, were not detected in the gene–spot expression profile; however, they demonstrated layer-specific APA usage in OPL and ONL, respectively. *Cep126* is a regulator of microtubule organization at the centrosome and has been identified to be associated with diseases such as monomelic amyotrophy [54]. Notably, even though the gene is not expressed in any spot, its poly(A) sites could be detected. This is probably because of the different tools or strategies used to quantify genes and poly(A) sites. Here, the gene expression profile was obtained using a conventional gene expression analysis pipeline, and the poly(A) site expression profile was obtained using scAPAtrap. scAPAtrap is considerably sensitive and can detect poly(A) sites for genes with low expression [25], whereas such genes may be undetectable using tools such as Cell Ranger. Owing to the fact that the sum of the expression levels of poly(A) sites in a gene can also be considered as the gene expression, we also summarized the read counts of all poly(A) sites in these two genes to represent their gene expression; however, no spatial pattern was observed for these two genes (Figure S3). These results indicate that the profile of APA usage encodes an additional layer of spatial information with high resolution that is invisible in the gene expression profile.

Next, we used SPARK to identify SVAPA genes. Using the imputed RUD matrix as the input, 133 genes with adjusted *P* value < 0.05 were considered SVAPA genes (Table S6). Similarly, we identified 772 SV genes with SPARK, using the gene–spot expression matrix as the input (Table S7). A considerably limited overlap was observed between these SVAPA and SV genes. Only 16 genes were detected as both SVAPA and SV genes (Figure S4), possibly because SVAPA and SV genes were detected based on APA and gene expression profiles, respectively. These two independent groups of genes possess spatial patterns at the gene and APA isoform levels.

**Figure 5:**
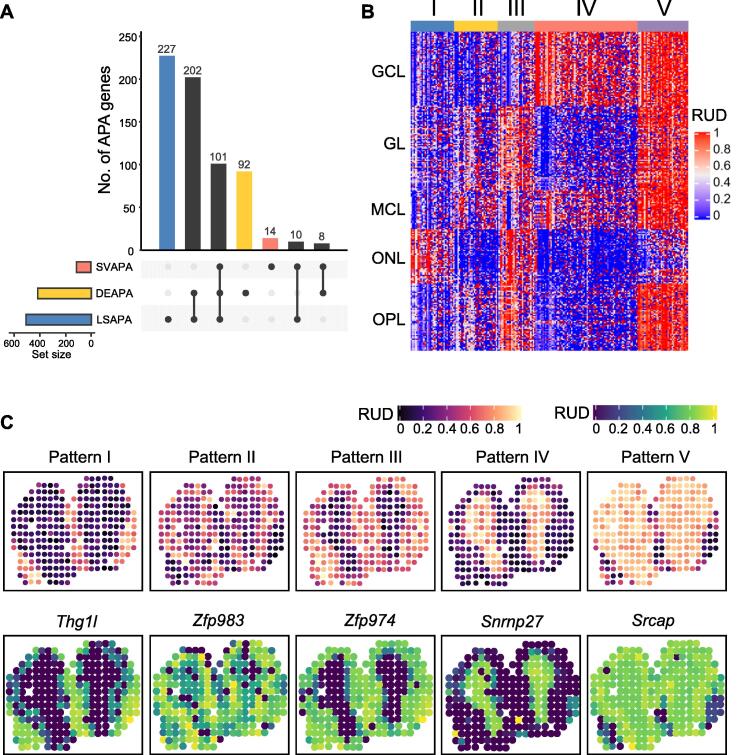
**Combined analysis of genes with spatial APA usage pattern** **A.** Upset plot showing overlap of DEAPA genes, LSAPA genes, and SVAPA genes. **B.** Five major spatial APA patterns by clustering on the combined gene set. **C.** Representative tissue images of the five patterns (top) and the corresponding example genes for individual patterns (bottom). The tissue image for each pattern was generated by averaging RUD scores on each spot for all genes with the respective pattern.

By combining DEAPA, LSAPA, and SVAPA genes, we obtained a comprehensive list of 654 non-redundant genes with spatial APA usage patterns (Figure 5A). A limited number of genes (101) were common to all three gene sets, indicating that these three gene sets are complementary in reflecting the full landscape of spatial APA dynamics. We performed clustering on the combined gene set and obtained the following five major spatial expression patterns (Figure 5B): pattern I representing ONL, pattern II representing the combination of GL and MCL; pattern III representing the combination of GL, ONL, and OPL; pattern IV representing GCL; and pattern V representing layers other than ONL. All five patterns were clearly visualized using five representative genes, namely *Thg1l*, *Zfp983*, *Zfp974*, *Srnp27*, and *Srcap* (Figure 5C). For example, *Thg1l*, encoding a tRNA-histidine guanylyltransferase 1-like protein associated with autosomal recessive ataxia with abnormal neurodevelopment [55], generally showed high RUD scores (*e.g.*, longer 3′ UTR) on ONL. In contrast, *Srcap*, encoding a *Snf2*-related CREBBP activator protein associated with diseases such as musculoskeletal defects and behavioral abnormalities, displayed higher RUD scores in multiple layers, except for ONL. Next, we performed functional enrichment analyses of the combined gene list of 654 genes. A total of 66 GO terms were enriched in these genes at a false discovery rate (FDR) of 5% (Table S8). Several enriched GO terms were directly associated with functions related to olfactory bulb development, such as synapse organization, neuron differentiation, and nervous system development. These APA genes and GO terms highlight the benefits of spatial APA analysis using stAPAminer.

### Spatial 3′ UTR APA patterns explored from additional replicates of ST-MOB demonstrate the robustness of stAPAminer

Next, we explored the spatial APA patterns from two additional replicates of ST-MOB to evaluate the robustness of stAPAminer. We extracted poly(A) sites from two adjacent tissue sections (Replicate 5 and Replicate 12) of ST-MOB for the analysis of spatial APA dynamics. The profile of poly(A) site expression of ST-MOB (Replicate 11) was highly similar to both replicates (Pearson’s correlation = 0.87 and 0.86 for Replicate 5 and Replicate 12, respectively) (Figure S5). Moreover, when repeating the imputation procedure on Replicate 5 (Figure S6) and Replicate 12 (Figure S7), we observed that our results were highly reproducible. For both replicates, different layers became more distinguishable after imputation (Figures S6A and S7A), and imputation improved spot separation or spot clustering (Figures S6B and S7B). These results demonstrate the robustness of the imputation method for stAPAminer. We also compiled DEAPA, SVAPA, and LSAPA genes for both replicates, resulting in 695 non-redundant genes for Replicate 5 (Table S9) and 556 non-redundant genes for Replicate 12 (Table S10). For all three replicates, considerably more LSAPA or DEAPA genes were identified than SVAPA genes (Figure 5A, Figure S8). This may be because different strategies were used to characterize spatial APA dynamics, *i.e.*, LSAPA or DEAPA genes were identified by comparing between layers, whereas SVAPA genes were identified by inferring the spatial trend globally.

**Figure 6:**
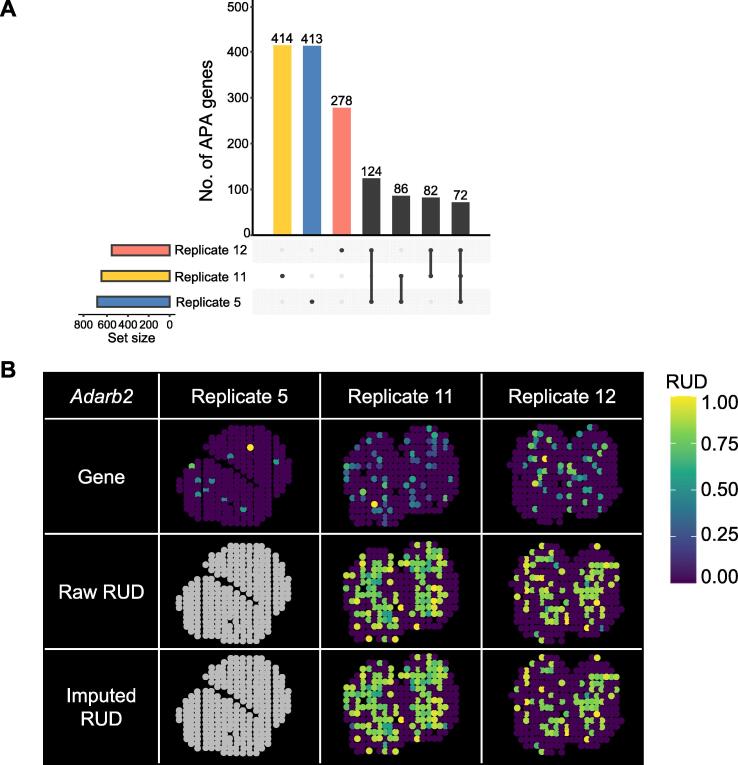
**Analysis of spatial APA dynamics from additional two replicates of ST-MOB with stAPAminer** **A.** Upset plot showing the overlap of genes with spatial APA pattern among the three replicates (Replicate 5, Replicate 11, and Replicate 12). **B.** An example gene (*Adarb2*) with differential APA usage between GCL and ONL. Tissue images based on the scaled gene expression level (top), raw RUD scores (middle), and imputed (bottom) RUD matrices were shown. Color represents RUD scores or scaled gene expression levels (yellow, high; blue, low).

Although the number of genes with spatial APA patterns was comparable among the three replicates, a limited number of consensus genes (364) were identified from two or more replicates, and a considerable number of genes were exclusively identified in one replicate (Figure 6A). The primary reason may be attributed to the inherent nature of the high sparsity and noise of the ST data, as well as the biological variance suffered from different replicates. Nevertheless, we observed several notable and reproducible results (Figures 6B, Figure S9). For example, DEAPA between GCL and ONL was observed for *Adarb2* in Replicate 11 (Figure 4C), and a similar pattern was also observed in Replicate 12 (Figure 6B), whereas this gene was not detected in Replicate 5. Notably, this gene also seemed to display a weak spatial gene expression pattern according to the tissue image; however, the pattern could not be detected by spatial expression analysis (*i.e.*, the gene is not present in Table S7). Similarly, *Mpp2*, enconding a postsynaptic MAGUK scaffold protein [56], showed a higher expression level or RUD score on GCL than other layers, and the pattern of APA usage was substantially more distinguishable than that of gene expression (Figure S9). *Mpp2* was also detected as an SV gene by spatial expression analysis (Table S7). In particular, the spatial pattern of APA usage was not observed because of the high dropout rate in Replicate 11, whereas the pattern was recovered after APA imputation and was highly consistent with that of Replicate 5. It is probable that some weak gene expression patterns may originate from noise, or some genes may possess both patterns of spatial gene expression and APA usage, but with varying strengths. We also observed some genes whose gene expression was not detected in the gene–spot matrix, although poly(A) sites and spatial APA patterns were detected in the imputed APA profile (*e.g.*, *Dnal1* and *Tmem268*) (Figure S9). This revealed the high sensitivity of stAPAminer in identifying and imputing APA profiles for genes that may be missed by conventional gene expression pipelines. These results demonstrate the ability and robustness of stAPAminer to identify spatial APA patterns.

### stAPAminer reveals extensive spatial dynamics of intronic APA usages from ST-MOB

**Figure 7:**
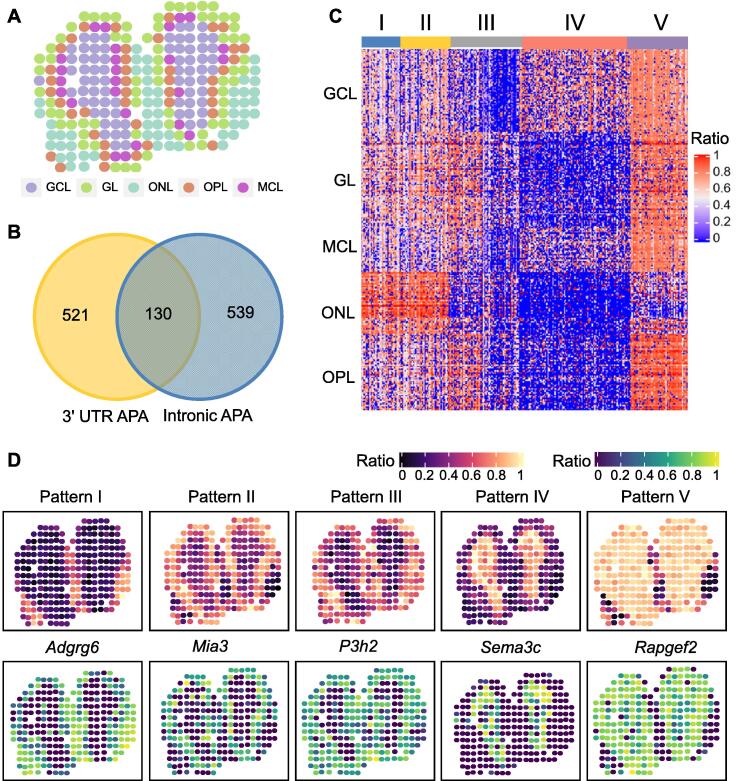
**Analysis of spatial patterns of intronic APA from ST-MOB** **A.** Visualization of ST spots on the tissue image after intronic APA imputation. **B.** Venn diagram showing the overlap of 3′ UTR APA genes and intronic APA genes with spatial APA patterns. **C.** Five major spatial APA patterns by clustering on the combined gene set. **D.** Representative tissue images of the five patterns (top) and the corresponding example genes for individual patterns (bottom). The tissue image for each pattern was generated by averaging intronic ratio scores on each spot for all genes with the respective pattern.

Having demonstrated the spatial dynamics of 3′ UTR APA, we next sought to explore the spatial patterns of intronic APA from ST-MOB. First, we obtained a matrix of intronic APA usage (see Materials and methods), containing 4011 genes present in 260 spots. The matrix was then imputed using the KNN model in stAPAminer (*k* = 10). The layer clustering result based on the imputed intronic APA signals was similar to that based on the 3′ UTR APA signal (ARI: intronic APA = 0.605, 3′ UTR APA = 0.643) (Figure 3B and Figure 7A). Based on the imputed intronic APA signal matrix, we further identified 601 non-redundant DEAPA genes (Table S11), 386 LSAPA genes (Table S12), and 226 SVAPA genes (adjusted *P* value < 0.05) (Table S13). Combining these three sets of genes, 669 non-redundant genes with spatial APA usage patterns were obtained, among which 130 genes showed spatial 3′ UTR APA patterns as well (Figure 7B). Next, we clustered these genes based on their intronic APA profiles and obtained the following five major spatial APA patterns (Figure 7C): pattern I representing ONL; pattern II representing the combination of GL and MCL; pattern III representing the combination of GL, ONL, and OPL; pattern IV representing GCL; and pattern V representing layers other than ONL. These five patterns were clearly visualized using five representative genes, namely *Adgrg6*, *Mia3*, *P3h2*, *Sema3c*, and *Rapgef2* (Figure 7D). *Adgrg6* is a protein-coding gene essential for the normal differentiation of promyelinating Schwann cells and normal myelination of axons [57]. *Mia3* is involved in cell migration related to sprouting angiogenesis [58]. *P3h2* encodes an enzyme catalyzing the post-translational formation of 3-hydroxyproline in collagens [59]. *Sema3c* encodes a protein which acts as an attractant for growing axons and thus plays a critical role in axonal outgrowth and guidance [60]. *Rapgef2* is involved in cAMP-induced Ras and Erk1/2 signaling, leading to sustained inhibition of long-term melanogenesis by reducing dendritic elongation and melanin synthesis [61]. Functional enrichment analysis of the combined gene list revealed that these genes were associated with the olfactory system, and most were directly related to the structure and regulation of synaptic organization (Table S14). In contrast, although genes with spatial 3′ UTR APA patterns were also associated with olfactory bulb development, they were enriched in synaptic organization, neuronal differentiation, and nervous system development (Table S8). These results indicate extensive spatial dynamics of intronic APA usage, even though genes with spatial intronic APA patterns are distinct from those with spatial 3′ UTR APA patterns.

### The KNN-based imputation model in stAPAminer is robust to data with different dropout rates and spot sizes

**Figure 8:**
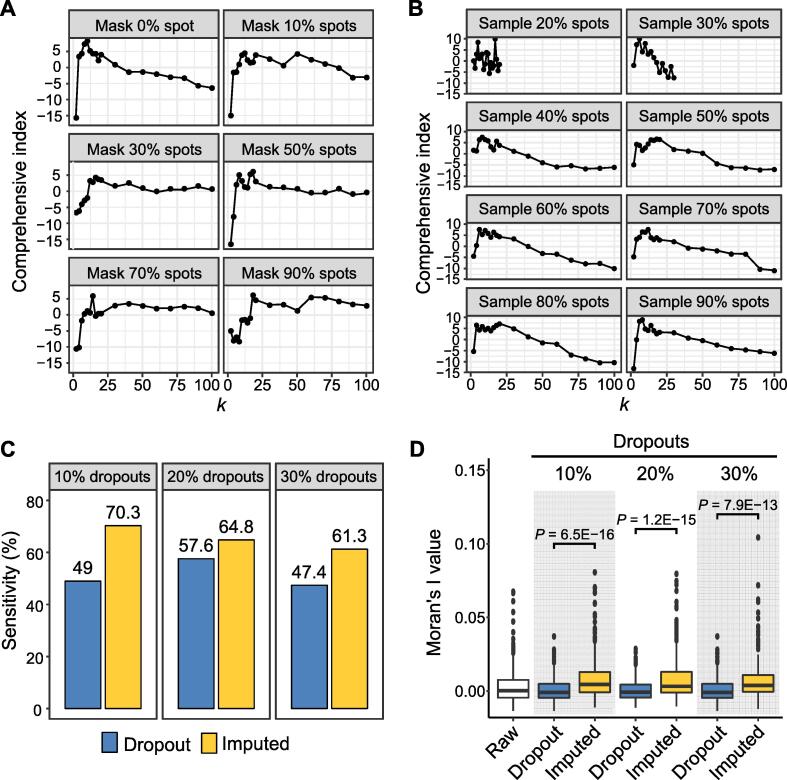
**Evaluation of the KNN-based imputation model using RUD matrix with varied spot sizes and dropout rates** **A.** The value of the comprehensive index with the increase in the *k* value under different number of dropout spots. The percentages of dropout spots were set from 10% to 90% by masking all genes in randomly selected spots, while keeping the spot number among layers unchanged. **B.** The value of the comprehensive index with the increase in the *k* value under different number of sampled spots. 20% to 90% spots were randomly sampled, while keeping the proportion of spot number among layers unchanged. **C.** Sensitivity of the KNN-based imputation model under RUD matrices with different dropout rates. The dropout rate of the raw RUD matrix was increased by 10%–30% through randomly masking values in the matrix. Genes with spatial APA pattern present in at least two replicates of Replicate 5, Replicate 11, and Replicate 12 were used as the true reference. Spatial APA patterns from the RUD matrix before and after imputation were identified to obtain sensitivity, respectively. **D.** Moran’s I value of spatial APA patterns identified from RUD matrices with different dropout rates. The RUD matrix was processed as in (C). For all plots in this figure, the RUD matrix of MOB Replicate 11 was used, which contained a total of 260 spots.

The KNN-based imputation method was embedded in our stAPAminer package to recover the sparse APA usage matrix; and the most critical parameter in the model was the *k* value. Here, we evaluated the influence of the spot size of the RUD matrix on the choice of the *k* value. First, we examined the influence of the number of dropout spots on the *k* value. We set the percentage of dropout spots in ST-MOB from 10% to 90% by masking all genes in randomly selected spots, while keeping the spot number among layers unchanged. Subsequently, the clustering performance based on the comprehensive index was evaluated under different *k* values at each dropout rate. In general, the larger the dropout rate, the larger the optimal *k* value (Figure 8A). This may be because when the dropout rate increases, more adjacent spots are needed to obtain sufficient information to achieve comparable clustering performance. However, even with an extremely high dropout rate (*e.g.*, > 50%), a *k* value within 20 can yield moderately high performance. Next, to evaluate the influence of the spot size on the *k* value, we randomly sampled 20% to 90% of spots from a total of 260 spots, while keeping the proportion of spot numbers among layers unchanged, and then tested the performance at different *k* values. Regardless of the spot size, the optimal *k* value was approximately 10 (Figure 8B). After reaching the optimal *k* value, the performance decreases gradually with the increase in *k* value, which may be because a large *k* value may result in increased biases by introducing the information of spots from other layers. In general, our model is robust to different *k* values. It is recommended to use a smaller *k* value with an equally high comprehensive index score to ensure high performance and avoid introducing biased information on spots from other layers owing to a large *k* value.

Next, we examined whether the KNN model in stAPAminer may be overfitted and evaluated the sensitivity of stAPAminer in recovering spatial APA patterns. To the best of our knowledge, this is the first study on the analysis of spatial APA patterns, and there is no gold standard for genes with spatial APA patterns; thus, it is difficult to validate whether an identified spatial APA pattern is true. Alternatively, we compiled a silver standard dataset containing genes with reproducible spatial APA patterns. Briefly, we first identified genes with spatial APA patterns in each of the three ST-MOB replicates (Replicate 5, Replicate 11, and Replicate 12) and established a list of 401 genes with spatial APA patterns present in at least two replicates. These genes were considered authentic instances and were used as true references for further evaluation. Assuming that our KNN-based imputation model is potentially overfitted, the accuracy of the spatial APA patterns identified from the RUD matrix with a higher dropout rate after imputation would be lower than that of the raw RUD matrix. Accordingly, we mimicked data with higher sparsity to verify whether the KNN-based imputation model is overfitted. We increased the dropout rate of the already sparse raw RUD matrix by 10%–30% by randomly masking the values in the matrix. We then used the KNN-based model to recover the extremely sparse RUD matrix. We identified spatial APA patterns from the RUD matrix before and after imputation and calculated the sensitivity according to the compiled silver standard. Regardless of the sparsity of the data, the sensitivity of pattern recognition on the imputed data was considerably higher than that before imputation (Figure 8C). Notably, the silver standard data that we constructed only represents true instances, whereas spatial APA patterns that are not in the silver standard are not necessarily false. Considering that there is no gold standard for false instances, only true positives (TP) and false negatives (FN), rather than false positives (FP) or true negatives (TN), can be calculated. Therefore, we calculated only the sensitivity to evaluate the results. Moreover, we used Moran’s I [62] value to measure the significance of the identified spatial patterns and observed that the spatial autocorrelation of the patterns obtained after imputation was significantly improved (Figure 8D). These results are substantially strong, even for highly sparse data, suggesting that our KNN-based imputation model is unlikely to be overfitted and can effectively enhance or recover the RUD signal, thereby considerably improving the sensitivity of spatial pattern recognition.

## Discussion

ST technologies have the advantage of revealing an unbiased map of transcripts in complex tissues and cell cultures. Established spatially resolved approaches can detect genes with localized expression patterns at a single-cell resolution. However, these approaches have primarily focused on gene-level analysis rather than isoform-level analysis. Consequently, these studies could neither measure isoform usage in a spatially defined tissue region nor detect significant spatial isoform usage in a spatial context. Therefore, it is essential to develop new methods for exploring isoform usage from ST data. The rich data generated by ST experiments promises isoform-level analysis, which complements conventional gene-level studies using ST data. In this study, we presented stAPAminer, a novel method for analyzing spatial APA dynamics in spatially resolved transcriptomic studies. The method allows to anchor the APA isoform usage in a spatial view and reveals crucial spatial expression patterns at isoform resolution. The integration of APA isoform usage and gene expression in a spatial context promises to establish spatial maps of APA dynamics. Our study further defines an atlas of APA events with distinct spatial APA usage patterns, which supplements SV genes to capture the spatial complexity of MOB subregions. We verified the identified genes with significant spatial APA usage in various ways and demonstrated the improvement of layer classification by integrating spatial APA information.

Limited by the sequencing depth, the gene expression profile obtained from ST data is usually sparse, with ubiquitous low counts and zeros. Moreover, the obtained APA profile is sparser than the already sparse gene expression profile, and this presents a huge computational challenge for studying the spatial patterns of APA isoform usage. The stAPAminer pipeline incorporates a KNN-based imputation model for recovering APA signals, thus mitigating the sparsity of the APA isoform matrix and yielding stronger spatial patterns. Evidence for APA isoform addition to cellular diversity has been bolstered by APA signal imputation (Figure 3). After imputation, the signals of genes with a spatial pattern of APA usage were retained or considerably enhanced (Figure 3F). Additional analyses using data with varied spot sizes and different numbers of dropout spots and genes demonstrated that the KNN model is robust to different *k* values and can substantially improve the sensitivity of spatial pattern recognition (Figure 8). stAPAminer has several desirable features for mitigating the high sparsity of APA profiles. First, the scAPAtrap [25] used in our pipeline can accurately identify all potential poly(A) sites, including those with low read coverage, by incorporating the strategies of peak identification and poly(A) read anchoring. Second, the KNN-based imputation model in stAPAminer can efficiently impute missing values by clustering spots based on gene signatures rather than the APA profile alone. This promises the inference of associations between ST spots by borrowing information from the gene expression profile of the ST data, thus avoiding the lack of information or overfitting when using the APA data alone. Third, the multiple rounds of iterations performed during the imputation process enable the gradual inclusion of newly imputed information. However, despite the effectiveness of stAPAminer in exploring spatial APA dynamics, it can be further improved in two aspects in the future. First, it is crucial to determine whether an entry into the APA ratio matrix is truly missing before imputation. Second, to identify global spatial patterns of APA, the APA ratio matrix is transformed into a count matrix to meet the input requirement of SPARK; such a transformation may not be theoretically reasonable or fully meet the model’s assumption. Additional work is required to develop more sophisticated approaches to address these issues.

Using stAPAminer, we identified three types of genes with spatial APA dynamics, namely DEAPA, LSAPA, and SVAPA genes. Moreover, we explored the spatial patterns of both the 3′ UTR APA and intronic APA. A total of 654 non-redundant 3′ UTR APA genes from the combined list of DEAPA, LSAPA, and SVAPA genes were obtained from the ST-MOB data (Figure 5A), representing five major spatial expression patterns (Figure 5B). Similarly, 601 intronic APA genes with spatial patterns were obtained; however, these genes were distinct from those with spatial 3′ UTR APA patterns (Figure 7). We provided several lines of evidence to validate these genes with spatial APA dynamics. First, the poly(A) sites identified from the ST-MOB data had high confidence, were supported by annotated poly(A) sites, and possessed typical poly(A) signals (Figure 2). Second, we examined the presence of these genes in the list of representative biologically important genes in the olfactory system from several previous studies [30], [36] (Table S4). Third, the enriched GO terms of these APA genes were directly associated with functions related to olfactory bulb development (Table S8). Fourth, when overlaying the APA signal of an APA gene on the tissue images, well-defined spatial patterns were revealed (Figure 3, Figure 4, Figure 5). Fifth, the spatial APA patterns of many genes were reproducible among the replicates (Figure 6, Figure S9). Notably, genes with spatial APA dynamics identified using stAPAminer are not necessarily present in genes obtained from conventional gene expression studies; however, these lines of evidence provide significant clues to the functional importance of the identified APA genes.

Compared with spatial patterns obtained using spatial expression analysis, we observed several notable cases of spatial APA patterns using stAPAminer. We identified some highly expressed genes with distinguishable spatial APA patterns but without any spatial gene expression patterns (*e.g.*, *St8sia5*, *Myef2*, and *Per1*) (Figure 3F). In addition, some genes presented detectable spatial APA and gene expression patterns, with the APA pattern being more distinguishable (*e.g.*, *Mpp2*) (Figure S9). Some genes, which seem to have spatial gene expression patterns but were not computationally detected using existing tools, demonstrated apparent spatial APA patterns (*e.g.*, *Adarb2*) (Figure 6B). These different groups of genes suggest the presence of SV patterns at the APA isoform level independent of the gene expression profile. In particular, some genes were not observed in the gene–spot matrix, whereas their spatial APA patterns were detected (*e.g.*, *Dnal1* and *Tmem268*) (Figure S9). This result is unlikely to be due to noise or random bias because we observed the same pattern in at least two replicates. The primary reason may be the different pipelines used to obtain the gene–spot expression matrix and the gene–spot RUD matrix. The scAPAtrap tool [25] used in this study has been proven to be highly sensitive and can identify poly(A) sites in extremely low-expressed genes that may not be detected in conventional gene expression analysis pipelines. Moreover, the APA signals are amplified through the imputation process of the stAPAminer, which further contributes to the successful identification of spatial APA patterns. These results demonstrate the effectiveness and robustness of stAPAminer in identifying different spatial APA patterns. In the future, it will be necessary to benchmark different methods for obtaining gene expression matrices and/or RUD matrices, as well as to propose methods that can mitigate discrepancies among replicates to identify more reproducible spatial patterns.

In summary, we present a detailed view of spatial APA usage across MOB regions using our proposed stAPAminer toolkit. To the best of our knowledge, our stAPAminer approach is one of the first computational approaches to explore the transcriptional atlas of spatial APA patterns with spatial resolution. stAPAminer employs the power of ST to explore genome-wide spatial patterns of APA usage variation at isoform resolution, establishing an additional layer of gene expression. The combination of spatial maps of gene expression and APA usage will allow us to deduce a more comprehensive set of genes summarizing spatial and cellular information and redefine the overall transcriptome complexity of a tissue.

## Materials and methods

### Data

The input of stAPAminer is a poly(A) site matrix, with each row being a poly(A) site and each column being a spot. Currently, there are several ST strategies such as MERFISH [28], seqFISH [29], and ST through spatial barcoding [30], among which APA sites can only be identified from the spatially barcoded ST data using existing scRNA-seq tools such as scAPAtrap or Sierra. However, if APA sites could be identified from other types of ST data in the future, stAPAminer would also be applicable to ST data. In this study, the spatially barcoded ST data of MOB [30] were used. We downloaded the gene expression measurements of the MOB data from Spatial Transcriptomics Research (https://www.spatialresearch.org/), which were collected from spatial locations known as spots. Corresponding array oligonucleotides with positional barcodes were also obtained. Following the studies of SpatialDE [31] and SPARK [33], the MOB Replicate 11 file was primarily used, which consisted of 16,218 genes measured on 262 spots. We retained spots with ten or more read counts, resulting in 260 spots. Two additional replicates of MOB (Replicate 5 and Replicate 12) were used for validation. Raw sequencing data were downloaded from Sequence Read Archive (SRA) of the National Center for Biotechnology Information (NCBI) (SRA: SRR3382373), which are publicly accessible at https://www.ncbi.nlm.nih.gov/sra. The lengths of barcodes and unique molecular identifiers (UMIs) were 18 nt and 9 nt, respectively. The genome assembly (version GRCm38) and latest genome annotation of mice were downloaded from Ensembl (https://www.ensembl.org).

### Identification and quantification of APA sites from ST data

The process of extracting APA sites from ST data was similar to that of scRNA-seq data. The raw ST data were double-stranded, including read 1 and read 2. First, barcodes and UMIs were extracted from read 1, and umi_tools [63] was adopted to append the barcode and UMI information to the sequence header of read 2 to generate a new read 2 FASTQ file. Reads from the read 2 file were aligned to the reference genome using STAR [64], and only uniquely mapped reads were retained. After mapping, PCR duplicates were removed using the dedup function in umi_tools, and one read per UMI for each genomic coordinate remained. scAPAtrap [25] was used to identify poly(A) sites from these mapped reads. Poly(A) sites were annotated with information, such as genomic regions and genes, with the movAPA package [65] using the R annotation package “TxDb.Mmusculus.UCSC.mm10.knownGene”. Similar to previous studies [66], [67], [68], [69], the 3′ UTRs of annotated genes were extended by 1000 bp to recruit intergenic poly(A) sites, which may originate from authentic 3′ UTRs.

### Quantification and imputation of spatial APA usage

We calculated the RUD for each 3′ UTR APA gene from the ST data using the movAPAindex function in movAPA [65]. Briefly, only 3′ UTR APA genes that contained at least two poly(A) sites in the 3′ UTR were retained. The distal poly(A) site is the one that is farthest from the start codon among all the 3′ UTR sites. The RUD score of each gene is the ratio of the expression level of the distal poly(A) site to the sum of the expression levels of all poly(A) sites located in the 3′ UTR. The RUD value ranged between 0 and 1. A larger RUD value of a gene in a spot indicated higher usage of the distal poly(A) site of this gene in this spot (*i.e.*, 3′ UTR lengthening). If no poly(A) site was expressed in a gene for a given spot, then the respective RUD value was called a dropout. Finally, an RUD matrix was obtained for the ST dataset, where each row denoted a gene and each column denoted a spot.

To explore the spatial patterns of intronic APA, we filtered poly(A) sites that were supported by at least three reads and present in at least three spots, and retained genes with multiple poly(A) sites and at least one intronic poly(A) site. We calculated the ratio for each intronic poly(A) site as the expression level of the respective site to the sum of the expression levels of all the sites in the same gene. The highest ratio of intronic poly(A) site(s) was considered the intronic APA usage of a gene. This ratio was also between 0 and 1. A larger ratio of a gene in a spot indicated higher usage of the intronic poly(A) site of the gene in this spot.

Owing to the inherent technical issues of spatial RNA-seq and lower expression at the APA isoform level than at the gene level, the APA usage matrix (RUD or ratio) is substantially sparser than the gene–spot expression matrix. Although the APA usage matrix contains usage information at the APA isoform level and has a higher resolution than the gene expression matrix, it was obtained from APA genes alone; consequently, it does not store as many genes as the gene–spot expression matrix. To mitigate the high sparsity of the APA usage matrix, we designed a KNN-based imputation model and introduced the gene–spot expression matrix to obtain the correlation between spots to better impute the APA usage matrix. Given a gene–spot matrix *G* with n genes and m spots, the matrix was first scaled (including data centering and standardization). Next, the Euclidean distance between the two spots was calculated to obtain a spot–spot distance matrix D [Equation (1)]. Subsequently, a ranking matrix $\overset{‾}{D}$ could be obtained, which was a spot–spot matrix that stored the spot indexes for each spot in descending order according to the distance between all other spots and the respective spot. $\overset{‾}{D_{\mathrm{ij}}}$ represents the spot index of the *j*-th spot closest to the *i*-th spot.

(1) $$D_{\mathrm{ij}}=\sqrt{\overset{n}{\underset{t=1}{\sum }}{\left(G_{t,i}^{*}-G_{t,j}^{*}\right)}^{2}}\;(i\;\mathrm{or}\;j=1,2,\cdots ,m)$$

Here, $G_{i,j}^{*}$ is the scaled gene expression level of gene i in spot j.

Given an APA usage matrix R with h genes and m spots, first, the values for those genes not expressed in the gene–spot matrix G were set to 0. Next, for each spot, the nearest k spots (default k=10) were selected according to the ranking matrix $\overset{‾}{D}$. The nearest k spots for any spot i (*i.e.*, the *i*-th row in $\overset{‾}{D}$) are the first k columns of the *i*-th row in the ranking matrix. Then, for a gene with missing APA usage in the matrix R, the average APA usage of this gene in these l spots is calculated for the imputation [Equation (2)]. Notably, only spots with non-zero APA usage for the gene were counted.

(2) $$R_{\mathrm{ij}}^{*}=\frac{1}{L}\sum_{x\in \left|k\right|}R_{i\overset{‾}{D_{\mathrm{jx}}}}\;\;when\;R_{\mathrm{ij}}\;is\;missing$$

(i=1,2,⋯,h;j=1,2,⋯,m)

Here, $R_{\mathrm{ij}}^{*}$ is the imputed APA usage for gene i in spot j; |k| denotes the set of nearest k spots with APA usage for the respective gene; L is the number of spots in $\left|k\right|$.

After the first round of imputation, a small part of the APA usage values remained missing when the respective genes were not expressed in any of the k nearest spots. Subsequently, we repeated the imputation step [Equation (2)] and performed multiple iterations until there were no missing entries or no additional missing entries could be filled. In each iteration, the average value of the APA usage of neighboring spots was calculated according to the newly imputed APA usage matrix. In addition, in the stAPAminer package, users can set the maximum number of iterations, which stops when the number of iterations reaches the set value. If there are still missing entries after the entire iteration process, we can directly set their values to 0.

Several performance indicators were adopted to evaluate the KNN-based imputation model. First, we manually obtained the true layer where each spot was located by combining the spatial information of each layer with the hematoxylin & eosin-stained bright field image of MOB slices. The true labels of the layers were used as references. To examine whether the imputed APA usage matrix better reflects the true relationship between spots than the raw APA usage matrix, we first adopted the FindClusters function (resolution = 5) in Seurat [70] to cluster spots based on the APA usage matrix and used the following four metrics to evaluate the performance in the context of clustering: ARI, Jaccard, Purity, and NMI. The ARI score ranged from −1 to 1, and the scores of Jaccard, Purity, and NMI ranged from 0 to 1, with higher values reflecting better performance. In addition, four internal validation metrics, namely DBI [38], CH [39], SC [40], and Dunn index [41], were employed to quantitatively assess the consistency of a clustering structure, which was independent of clustering methods or prior knowledge of true labels. A smaller DBI score or higher CH, SC, or Dunn score indicated better separation among clusters. Moreover, we calculated Pearson’s correlation coefficient between each pair of spots under the same layer to examine whether the correlation of spots on the same layer after imputation was higher than that before imputation.

The most important parameter in our KNN-based imputation model was the *k* value. We proposed a strategy to determine the optimal *k* value based on a comprehensive index. Briefly, given a set of *k* values, the APA usage matrix is imputed for each *k*. Clustering was then performed on the imputed matrix to obtain eight clustering index values, including four internal validation metrics (DBI, SC, CH, and Dunn) and four external metrics (ARI, Jaccard, NMI, and Purity). Next, the Z-score was calculated for each index, and the sum of all Z-score values was considered the comprehensive index. Finally, the *k* value with the highest comprehensive index score was regarded as the optimal *k*.

### Identification of genes with spatial patterns of APA usage

To fully explore the spatial patterns of APA usage, we identified three types of APA genes, namely DEAPA, LSAPA, and SVAPA. DEAPA genes exhibit differential APA usage between the two layers and are similar to DEGs in conventional gene expression studies. LSAPA genes exhibit layer-specific APA usage. SVAPA genes exhibit distinct spatial APA usage patterns in the global spatial context, which are similar to SV genes in conventional ST studies. We followed the Seurat tutorial [70] to cluster spots based on the APA usage matrix and obtained five spot clusters (equal to the number of layers). These clusters were annotated as GCL, MCL, OPL, GL, and ONL according to the hematoxylin & eosin staining image. To identify DEAPA genes, we used the FindMarkers function in Seurat with the APA usage matrix as input. This function identifies DEAPA genes between two groups of spots using the Wilcoxon Rank Sum test. Genes with an adjusted *P* value < 0.05 and log_2_ fold change > 0.5 were considered DEAPA genes. We also identified LSAPA genes using FindMarkers by comparing spots in one layer to all spots in the remaining layers.

In addition, we detected genes with SV APA usages, *i.e.*, SVAPA genes. In contrast to DEAPA genes that show differential APA usage between the two groups of spots, SVAPA genes have distinct spatial APA usage patterns in the global spatial context. Currently, there is no tool available for identifying global spatial patterns from ratio-type data, but there are several tools for identifying SV genes, which can be applied to the APA usage matrix. SPARK [34] utilize a non-parametric method to effectively detect spatially expressed genes from large ST data, which controls type I errors and produces high power. SPARK is identified to be ten times more powerful than other existing methods [34]. Considering that SPARK is designed for count data, we transformed the APA usage values, which are between 0 and 1, into count data by taking 10 as the base (for 3′ UTR APA) or by log_2_ conversion (for intronic APA). This transformation made the data approximately conform to the Poisson distribution required by SPARK. The transformed APA usage matrix was then used as the input for SPARK, and genes with adjusted *P* value < 0.05 were considered genes with spatial APA usage patterns. Moreover, we implemented a unified interface in stAPAminer to import SVAPA results, which facilitates the incorporation of results from other tools upon the availability of more dedicated tools in the future.

After obtaining the DEAPA, LSAPA, and SVAPA genes, we combined these three sets of genes without redundancy to compile a unique gene set with dynamic APA usage in a spatial context. We then adopted k-means to cluster these genes into ten groups based on their APA usage profile, using the kmeans function of the R package stats with arguments “iter.max = 1e+9, nstart = 1000”. Next, the mean APA usage profile for each group of genes was calculated for each spot; each group was considered one spatial APA pattern. We selected five groups with the most distinguished spatial APA usage patterns. Moreover, we calculated Pearson’s correlation to measure the similarity between the APA usage profile of each gene in a group and the average profile of the group, and selected genes with Pearson’s correlation > 0.5 and *P* < 0.05, as representative genes with spatial APA patterns.

## Code availability

Our implementation of stAPAminer is available at https://github.com/BMILAB/stAPAminer and https://ngdc.cncb.ac.cn/biocode/tools/BT007320.

## Competing interests

The authors have declared no competing interests.

## CRediT authorship contribution statement

**Guoli Ji:** Investigation, Methodology, Data curation, Writing – review & editing. **Qi Tang:** Data curation, Software, Visualization, Writing – review & editing. **Sheng Zhu:** Data curation, Software, Visualization. **Junyi Zhu:** Data curation, Formal analysis. **Pengchao Ye:** Formal analysis. **Shuting Xia:** Formal analysis. **Xiaohui Wu:** Conceptualization, Writing – original draft, Writing – review & editing, Supervision, Project administration, Funding acquisition. All authors have read and approved the final manuscript. All authors read and approved the final manuscript.

## Supplementary material

Supplementary Figure S1 **Motifs identified in the upstream 50 nt region of the poly(A) site** Motifs were identified by MEME for 3′ UTR poly(A) sites. Top ten motifs, each with three most significant hexamers, were shown.

Supplementary Figure S2 **Scatter plot showing the correlation of poly(A) site expression profiles obtained from ST-MOB and bulk 3′ end sequencing (3′-seq)** Each dot is one poly(A) site and both axes are natural-log scaled. The Pearson’s correlation is indicated in the plot. Here the bulk 3'-seq data contains a total of 32 neural-related samples from the PolyASite 2.0 database.

Supplementary Figure S3 **Two representative genes showing clear spatial APA usage patterns but no gene expression pattern** The “Gene” row is the average gene expression level of the respective gene in all spots which represented by the sum of expression levels of poly(A) sites in the gene. The “RUD” row is the average RUD score of the gene in all spots.

Supplementary Figure S4 **Overlap between SVAPA genes and SV genes** The 16 overlapped genes are *Sft2d2*, *Runx1t1*, *Epha5*, *Gabrb2*, *Gja1*, *Igfbp4*, *Tmem132b*, *Fam149a*, *Camk1d*, *Ak5*, *Pde5a*, *Rbfox1*, *Crtc1*, *Grasp*, *Magt1*, *and Fibcd1*. SV, spatially variable.

Supplementary Figure S5 **Scatter plots showing expression levels of poly(A) sites between replicates A.** Correlation between Replicate 5 and Replicate 11. **B.** Correlation between Replicate 5 and Replicate 12. Each dot is one poly(A) site and both axes are log2 scaled.

Supplementary Figure S6 **Validation of stAPAminer in imputing APA signals using Replicate 5 of ST-MOB A.** Visualization of ST spots on the tissue image before (Raw) and after (Imputed) imputation. **B.** Evaluation of the performance of the imputation model. Four metrics were used for evaluating the performance in the context of clustering, including ARI, Jaccard, NMI, and Purity, and four internal validation metrics without relying on the reference labels were also used, including DBI, CH, SC, and Dunn. **C.** Boxplot showing Pearson’s correlations between spot pairs in each layer estimated using imputed and the raw RUD scores. For each layer, correlations of all pairwise spots were calculated.

Supplementary Figure S7 **Validation of stAPAminer in imputing APA signals using Replicate 12 of ST-MOB A.** Visualization of ST spots on the tissue image before (Raw) and after (Imputed) imputation. **B.** Evaluation of the performance of the imputation model. Four metrics were used for evaluating the performance in the context of clustering, including ARI, Jaccard, NMI, and Purity, and four internal validation metrics without relying on the reference labels were also used, including DBI, CH, SC, and Dunn. **C.** Boxplot showing Pearson’s correlations between spot pairs in each layer estimated using imputed and the raw RUD scores. For each layer, correlations of all pairwise spots were calculated.

Supplementary Figure S8 **Upset plot showing the overlap of DEAPA, LSAPA, and SVAPA genes A.** The result for Replicate 5. **B.** The result for Replicate 12.

Supplementary Figure S9 **Representative genes with spatial APA dynamics detected from the three replicates of ST-MOB** Tissue images of the respective gene based on the scaled gene expression level (top), raw RUD scores (middle) and imputed (bottom) RUD matrices were shown. Color represents RUD scores or scaled gene expression levels (yellow, high; blue, low).

Supplementary Table S1 **Poly(A) sites identified from ST-MOB using scAPAtrap**

Supplementary Table S2 **DEAPA genes between each pair of morphological layers**

Supplementary Table S3 **Differentially expressed genes identified using the gene–spot expression matrix**

Supplementary Table S4 **List of representative genes in the olfactory system presented in previous studies or public resources**

Supplementary Table S5 **LSAPA genes by comparing the RUD profile of a layer to all other layers**

Supplementary Table S6 **SVAPA genes identified by SPARK**

Supplementary Table S7 **SV genes identified by SPARK**

Supplementary Table S8 **GO terms enriched in the combined list of DEAPA, LSAPA, and SVAPA genes**

Supplementary Table S9 **DEAPA, LSAPA, and SVAPA genes identified for Replicate 5 of ST-MOB**

Supplementary Table S10 **DEAPA, LSAPA, and SVAPA genes identified for Replicate 12 of ST-MOB**

Supplementary Table S11 **DEAPA genes of intronic APA between each pair of morphological layers**

Supplementary Table S12 **LSAPA genes of intronic APA by comparing the intronic ratio profile of a layer to all other layers**

Supplementary Table S13 **SVAPA genes that display spatial intronic APA patterns identified by SPARK**

Supplementary Table S14 **Top 10 GO terms enriched in the combined gene list of DEAPA, LSAPA, and SVAPA genes with intronic APA**
