# Supplementary figures and images for: stAPAminer: Mining Spatial Patterns of Alternative Polyadenylation for Spatially Resolved Transcriptomic Studies
Source: Genomics Proteomics Bioinformatics. 2023 Jan 18;21(3):601–18. doi: 10.1016/j.gpb.2023.01.003 (PMC10787175; doi:10.1016/j.gpb.2023.01.003)

## Slide 1
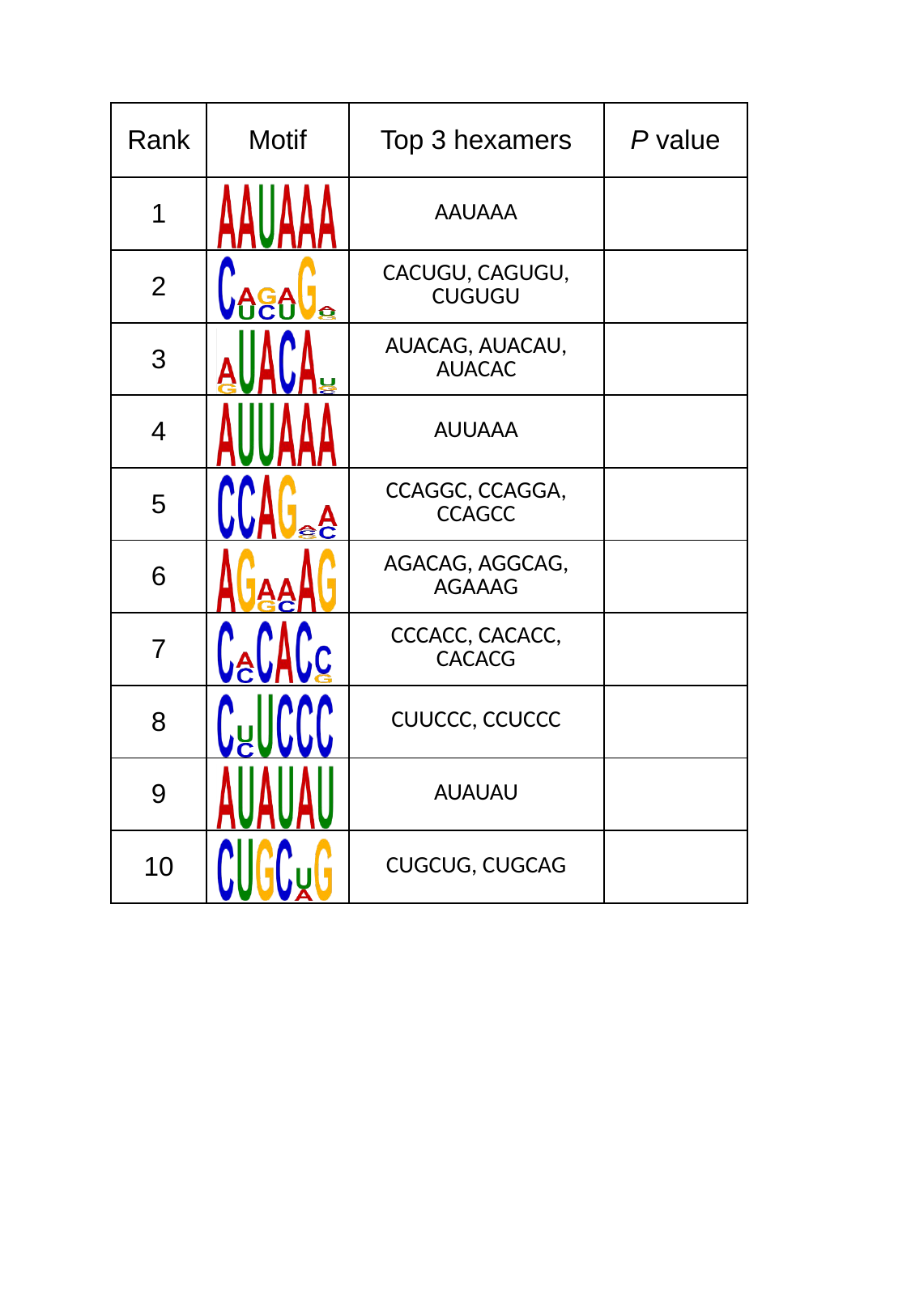

Supplement: Supplementary Figure S1 — Motifs identified in the upstream 50 nt region of the poly(A) site Motifs were identified by MEME for 3′ UTR poly(A) sites. Top ten motifs, each with three most significant hexamers, were shown. [file mmc1.pptx]

## Slide 1
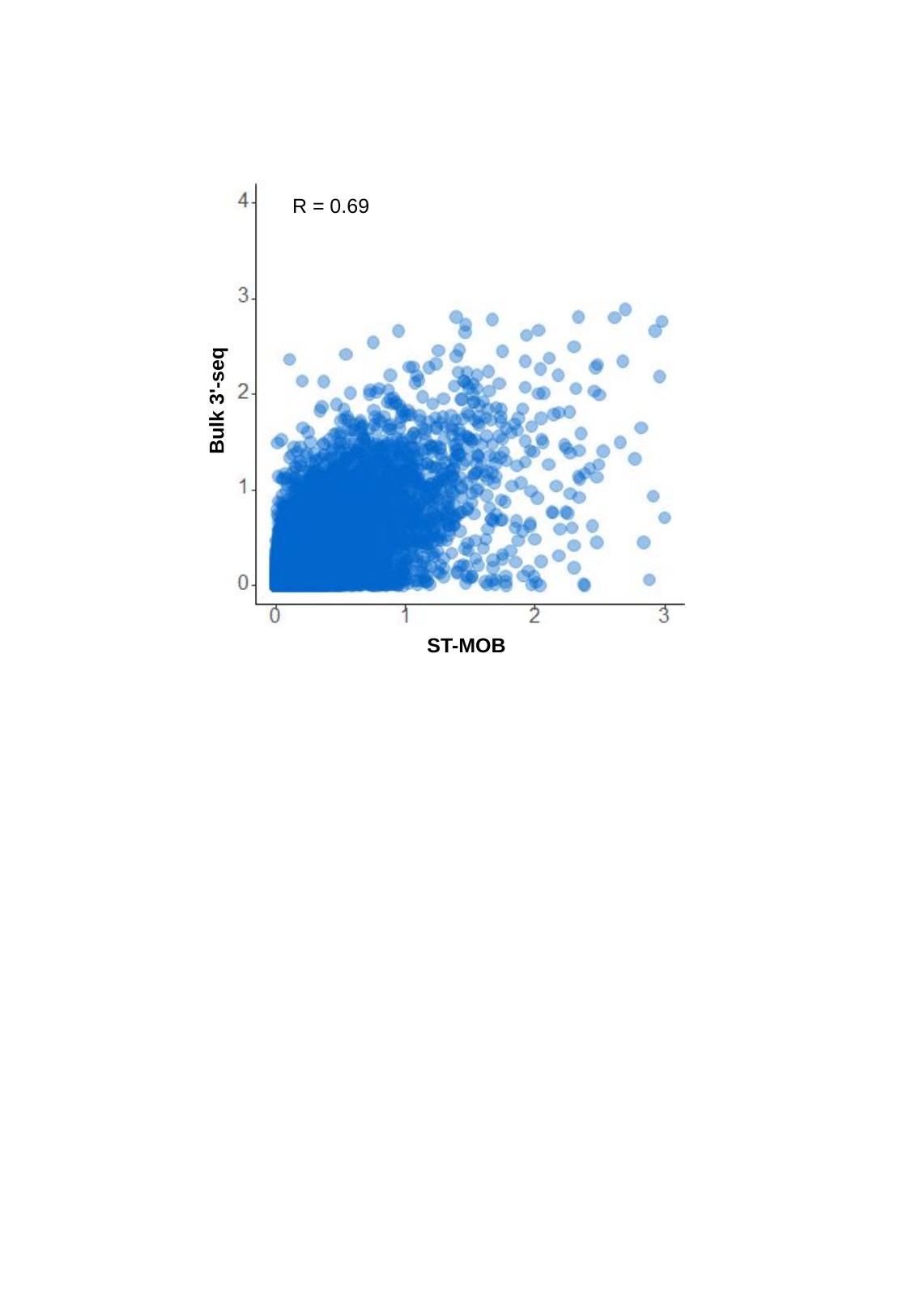

R = 0.69
Bulk 3'-seq
ST-MOB

Supplement: Supplementary Figure S2 — Scatter plot showing the correlation of poly(A) site expression profiles obtained from ST-MOB and bulk 3′ end sequencing (3′-seq) Each dot is one poly(A) site and both axes are natural-log scaled. The Pearson’s correlation is indicated in the plot. Here the bulk 3'-seq data contains a total of 32 neural-related samples from the PolyASite 2.0 database. [file mmc2.pptx]

## Slide 1
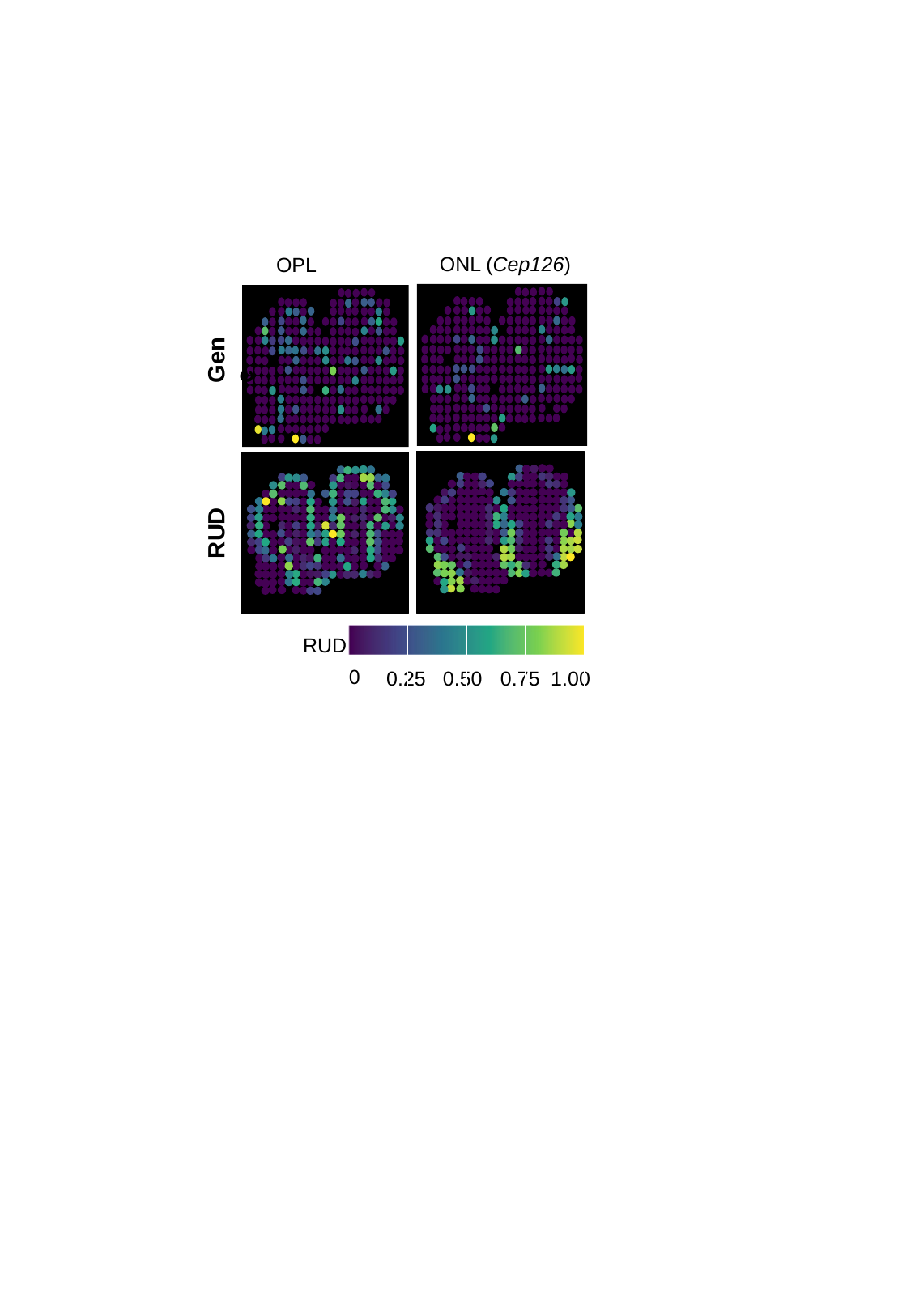

ONL (Cep126)
OPL (Dhdh)
Gene
RUD
1.00
0.75
0.50
0.25
0
RUD

Supplement: Supplementary Figure S3 — Two representative genes showing clear spatial APA usage patterns but no gene expression pattern The “Gene” row is the average gene expression level of the respective gene in all spots which represented by the sum of expression levels of poly(A) sites in the gene. The “RUD” row is the average RUD score of the gene in all spots. [file mmc3.pptx]

## Slide 1
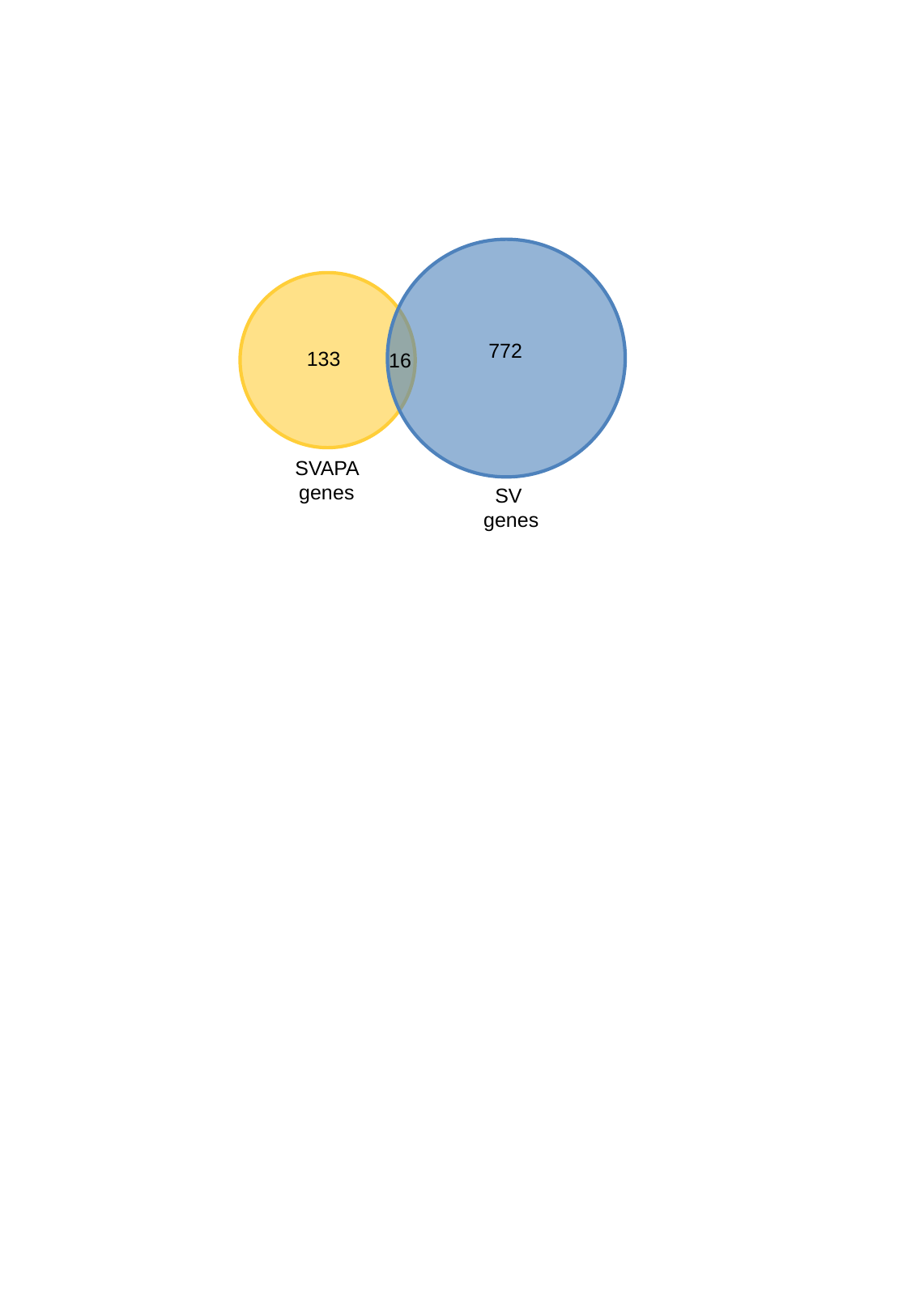

772
133
16
SVAPA genes
SV
genes

Supplement: Supplementary Figure S4 — Overlap between SVAPA genes and SV genes The 16 overlapped genes are Sft2d2, Runx1t1, Epha5, Gabrb2, Gja1, Igfbp4, Tmem132b, Fam149a, Camk1d, Ak5, Pde5a, Rbfox1, Crtc1, Grasp, Magt1, and Fibcd1. SV, spatially variable. [file mmc4.pptx]

## Slide 1
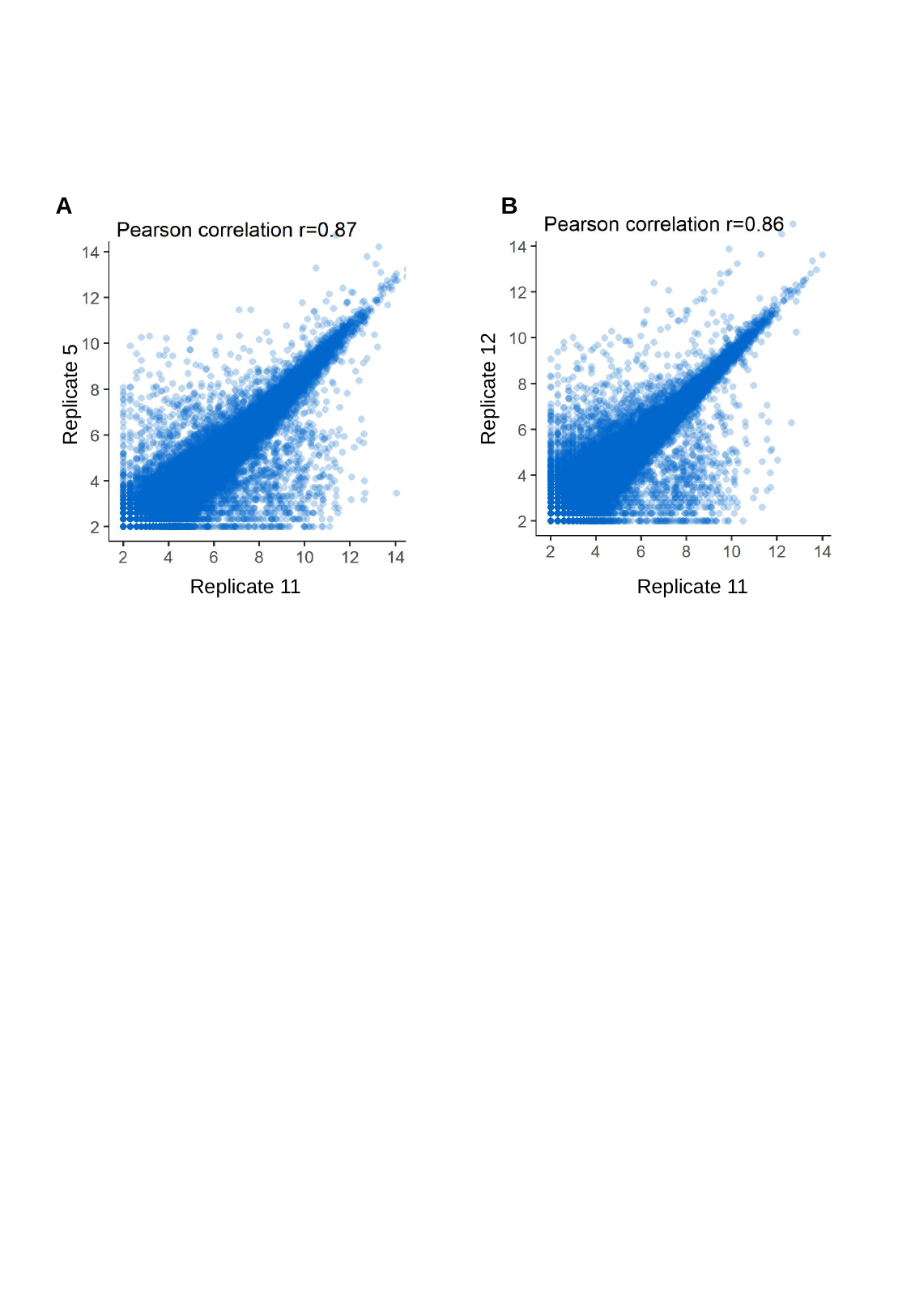

A
B
Replicate 5
Replicate 12
Replicate 11
Replicate 11

Supplement: Supplementary Figure S5 — Scatter plots showing expression levels of poly(A) sites between replicates A. Correlation between Replicate 5 and Replicate 11. B. Correlation between Replicate 5 and Replicate 12. Each dot is one poly(A) site and both axes are log2 scaled. [file mmc5.pptx]

## Slide 1
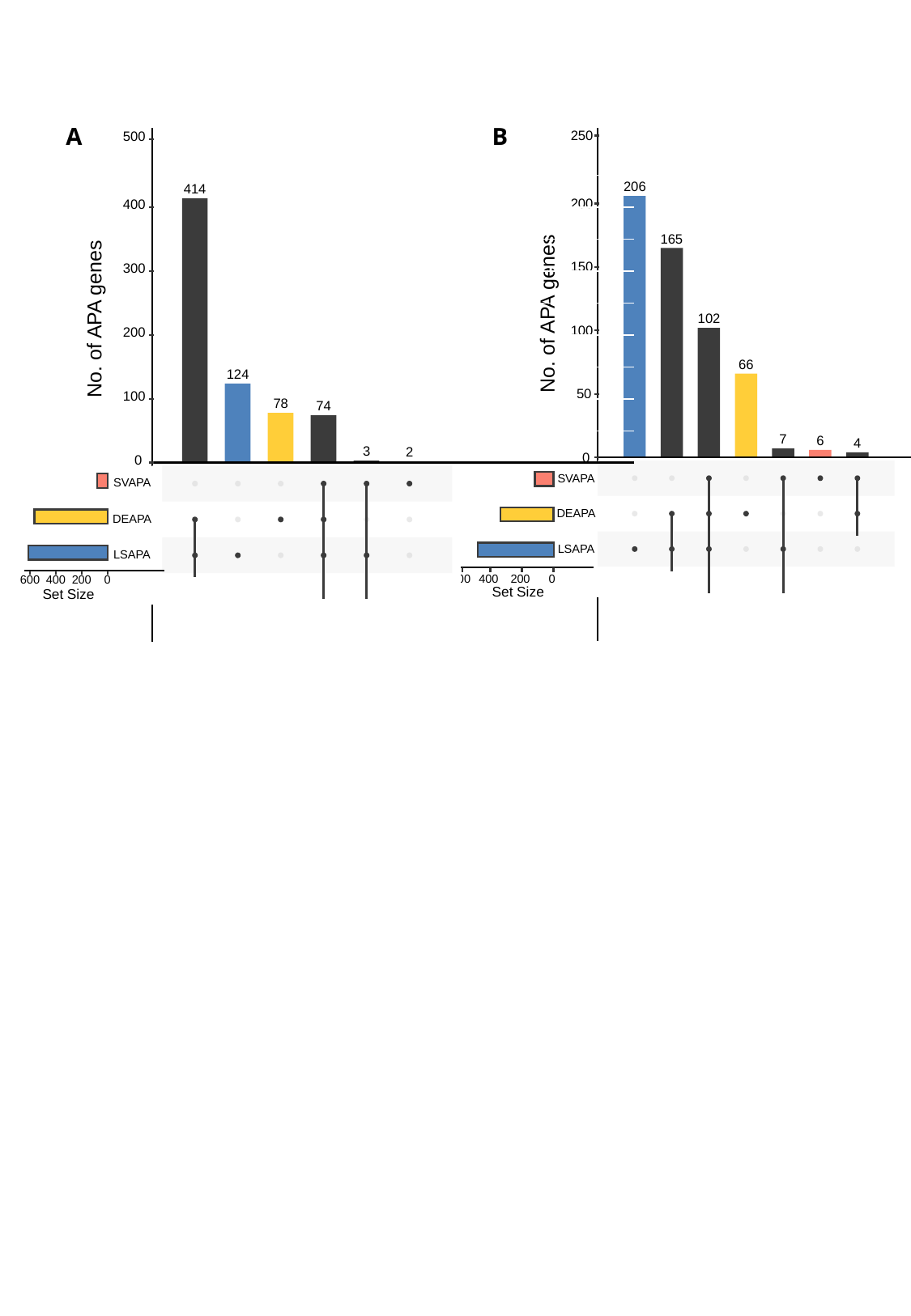

A
B
250
206
200
165
150
No. of APA genes
102
100
66
50
7
6
4
0
600
400
200
0
Set Size
 SVAPA
 DEAPA
 LSAPA
500
414
400
300
No. of APA genes
200
124
100
78
74
3
2
0
600
400
200
0
Set Size
 SVAPA
 DEAPA
 LSAPA

Supplement: Supplementary Figure S8 — Upset plot showing the overlap of DEAPA, LSAPA, and SVAPA genes A. The result for Replicate 5. B. The result for Replicate 12. [file mmc8.pptx]

## Slide 1
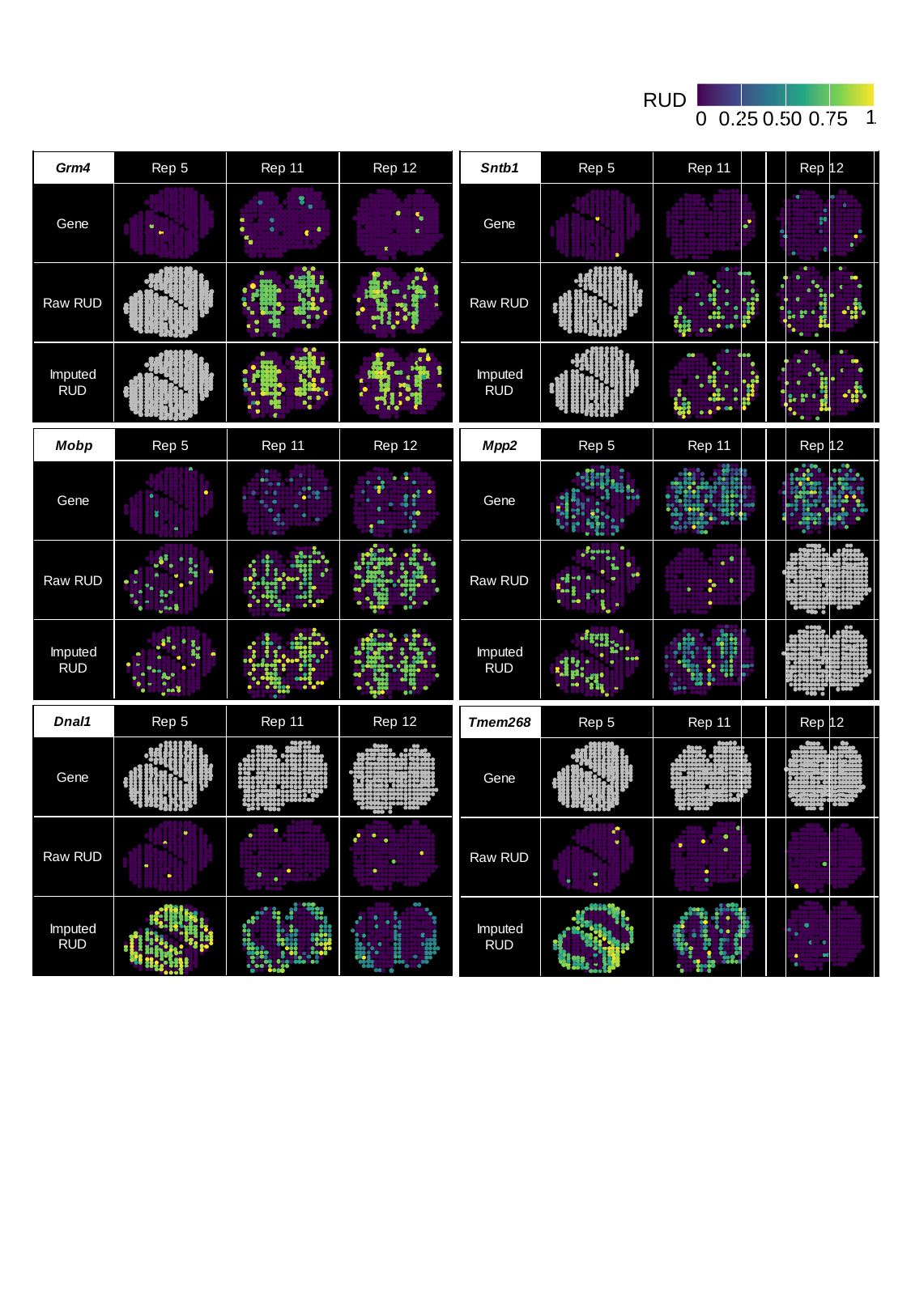

1
0.75
0.50
0.25
0
RUD

Supplement: Supplementary Figure S9 — Representative genes with spatial APA dynamics detected from the three replicates of ST-MOB Tissue images of the respective gene based on the scaled gene expression level (top), raw RUD scores (middle) and imputed (bottom) RUD matrices were shown. Color represents RUD scores or scaled gene expression levels (yellow, high; blue, low). [file mmc9.pptx]
