# Supplementary material for: stAPAminer: Mining Spatial Patterns of Alternative Polyadenylation for Spatially Resolved Transcriptomic Studies
Source: Genomics Proteomics Bioinformatics. 2023 Jan 18;21(3):601–18. doi: 10.1016/j.gpb.2023.01.003 (PMC10787175; doi:10.1016/j.gpb.2023.01.003)
Supplement: Supplementary Figure S6 — Validation of stAPAminer in imputing APA signals using Replicate 5 of ST-MOB A. Visualization of ST spots on the tissue image before (Raw) and after (Imputed) imputation. B. Evaluation of the performance of the imputation model. Four metrics were used for evaluating the performance in the context of clustering, including ARI, Jaccard, NMI, and Purity, and four internal validation metrics without relying on the reference labels were also used, including DBI, CH, SC, and Dunn. C. Boxplot showing Pearson’s correlations between spot pairs in each layer estimated using imputed and the raw RUD scores. For each layer, correlations of all pairwise spots were calculated. [file mmc6.pptx]

## Slide 1
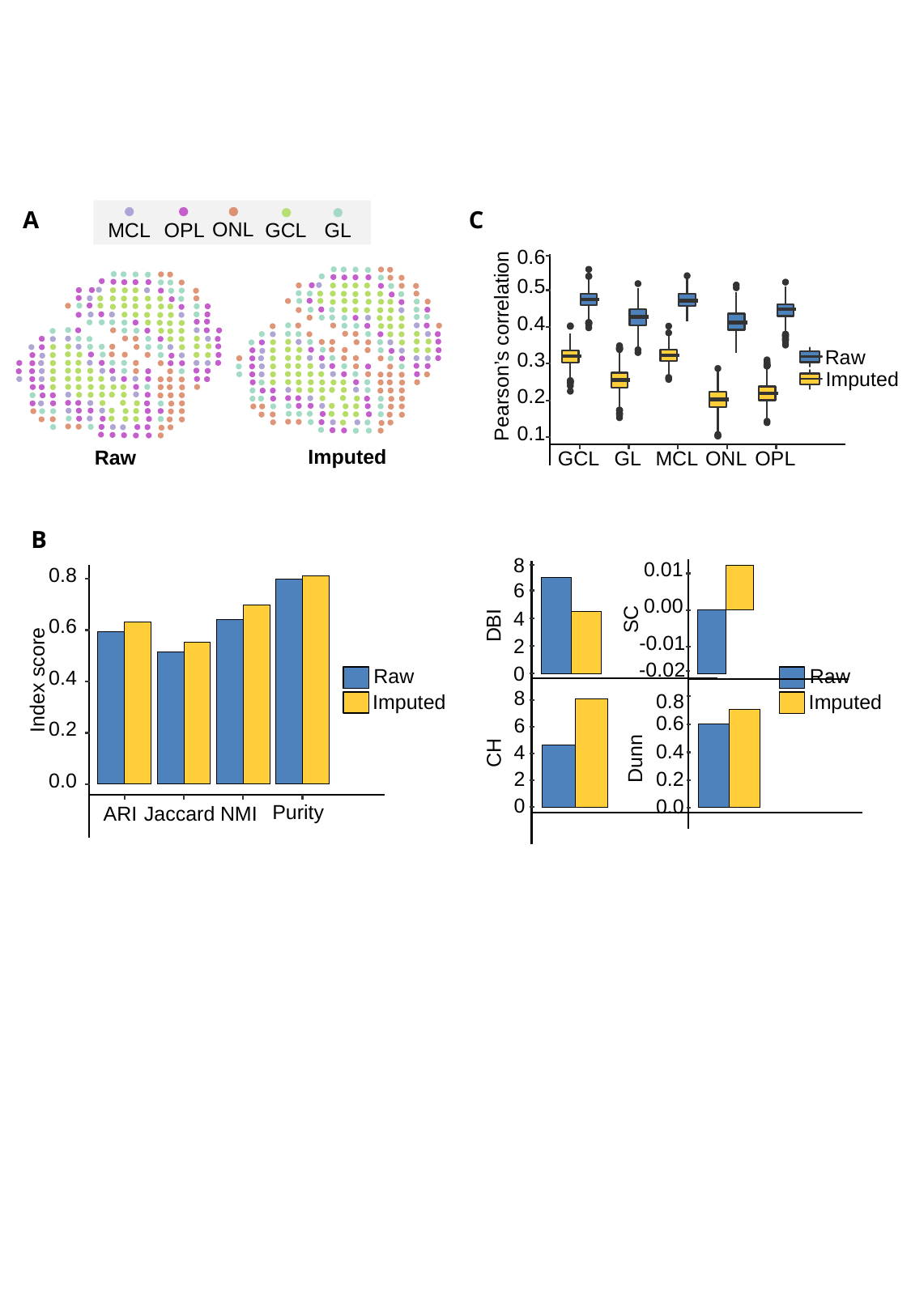

A
C
MCL
OPL
ONL
GCL
GL
Imputed
Raw
0.6
0.5
0.4
Pearson’s correlation
Raw
Imputed
0.3
0.2
0.1
ONL
OPL
GL
MCL
GCL
B
8
6
4
DBI
2
0
0.8
0.6
Raw
Imputed
Index score
0.4
0.2
0.0
Purity
Jaccard
ARI
NMI
0.01
0.00
SC
-0.01
Raw
Imputed
-0.02
0.8
0.6
0.4
Dunn
0.2
0.0
8
6
CH
4
2
0
